# Deciphering rhizosphere microbiome assembly of wild and modern common bean (*Phaseolus vulgaris*) in native and agricultural soils from Colombia

**DOI:** 10.1186/s40168-019-0727-1

**Published:** 2019-08-14

**Authors:** Juan E. Pérez-Jaramillo, Mattias de Hollander, Camilo A. Ramírez, Rodrigo Mendes, Jos M. Raaijmakers, Víctor J. Carrión

**Affiliations:** 1 Department of Microbial Ecology, Netherlands Institute of Ecology (NIOO-KNAW), P.O. Box 50, Wageningen, 6708 PB The Netherlands; 2 Institute of Biology, Leiden University, Sylviusweg 72, Leiden, 2333 BE The Netherlands; 3 Institute of Biology, University of Antioquia, Calle 67 #53-108, Medellín, Colombia; 4 Embrapa Meio Ambiente, Rodovia SP 340 - km 127.5, Jaguariúna, 13820-000 Brazil

**Keywords:** Common bean, Wild and modern accessions, Domestication, Rhizosphere, Core microbiome, Networks

## Abstract

**Background:**

Modern crop varieties are typically cultivated in agriculturally well-managed soils far from the centers of origin of their wild relatives. How this habitat expansion impacted plant microbiome assembly is not well understood.

**Results:**

Here, we investigated if the transition from a native to an agricultural soil affected rhizobacterial community assembly of wild and modern common bean (*Phaseolus vulgaris*) and if this led to a depletion of rhizobacterial diversity. The impact of the bean genotype on rhizobacterial assembly was more prominent in the agricultural soil than in the native soil. Although only 113 operational taxonomic units (OTUs) out of a total of 15,925 were shared by all eight bean accessions grown in native and agricultural soils, this core microbiome represented a large fraction (25.9%) of all sequence reads. More OTUs were exclusively found in the rhizosphere of common bean in the agricultural soil as compared to the native soil and in the rhizosphere of modern bean accessions as compared to wild accessions. Co-occurrence analyses further showed a reduction in complexity of the interactions in the bean rhizosphere microbiome in the agricultural soil as compared to the native soil.

**Conclusions:**

Collectively, these results suggest that habitat expansion of common bean from its native soil environment to an agricultural context had an unexpected overall positive effect on rhizobacterial diversity and led to a stronger bean genotype-dependent effect on rhizosphere microbiome assembly.

**Electronic supplementary material:**

The online version of this article (10.1186/s40168-019-0727-1) contains supplementary material, which is available to authorized users.

## Background

Plant domestication and the agricultural revolution provided a more continuous food supply to early human hunter-gatherer groups and were key drivers of the conformation of stable human settlements [1]. Domestication led to major changes both in phenotypic and genotypic traits of crop varieties including larger seed size, loss of dispersal mechanisms, and determinate growth [2, 3]. However, domestication also led to a reduction in genetic diversity, referred to as the domestication syndrome [2]. Recent studies further showed that domestication affected rhizosphere microbiome assembly. Plants rely, at least in part, on their rhizosphere microbiome for functions and traits related to nutrient acquisition, enhanced stress tolerance, protection against soil-borne pathogens, and host immune regulation [4, 5]. For several plant species [6], including sugar beet [7], barley [8], sunflower [9], and common bean [10], differences in rhizosphere microbiome composition between wild relatives of crop plants and their domesticated counterparts have been observed. For common bean, we previously revealed that wild accessions were enriched in bacterial taxa from the phylum *Bacteroidetes*, whilst modern accessions were enriched in *Actinobacteria* and that this compositional shift was associated with plant genotypic as well as root phenotypic traits [10].

Plant domestication not only comes with changes in plant traits, but is also accompanied by progressive changes in the habitat and crop management practices to promote high yields and to protect the domesticated plants from biotic and abiotic stress factors [11]. Hence, the transition from native habitats to agricultural soils may have led to a depletion of plant-associated microbes thereby affecting specific, co-evolved beneficial functions of the plant microbiome. For example, long-term nitrogen fertilization resulted in the evolution of less mutualistic rhizobia, providing fewer benefits to the host [12]. Similarly, it was shown that nitrogen amendments suppressed soil respiration and microbial biomass, promoting copiotrophs such as *Actinobacteria* and *Firmicutes* while reducing the abundance of oligotrophs such as *Acidobacteria* and *Verrucomicrobia* [13]. It has been also shown that the occurrence of members of the phylum *Bacteroidetes*, whose members are known for their abilities to degrade complex polymeric organic matter, is negatively affected by agricultural soil management practices [14]. Moreover, conversion of the Amazon rainforest to agriculture resulted in biotic homogenization of soil bacterial communities and a net loss of microbial diversity [15]. For most crop plants, however, there is little knowledge on the co-evolutionary trajectory between plants and their microbiomes during habitat expansion and if domestication indeed led to a reduced microbial diversity and a depletion of specific microbial genera in these new habitats.

In this study, we used *Phaseolus vulgaris* (common bean) as “model” plant species. Common bean originated in central Mexico and as a wild species spread throughout Central and South America [16–18]. The geographic isolation of wild common bean resulted in the establishment of the Mesoamerican and Andean genetic pools [19] which were the basis of two independent domestication processes. As a consequence, domesticated common bean underwent several morphological and physiological changes as well as a significant reduction in genetic diversity [20–22]. We selected wild, landraces, and modern accessions of Mesoamerican common bean originating from Colombia based on a number of genetic and phenotypic traits [10]. Here, we hypothesized that the transition of common bean from a native soil environment to an agriculturally managed soil led to a depletion of bacterial diversity in the rhizosphere microbiome. We further hypothesized that this depletion of rhizobacterial diversity is stronger for domesticated common bean accessions than for their wild relatives. To address these hypotheses, eight common bean accessions, representing a trajectory from wild to modern [10], were grown in a native soil and in an agricultural soil collected in the Colombian highlands, followed by rhizobacterial community profiling, species abundance modeling, and co-occurrence network analyses.

## Material and methods

### Selection of soils and plant accessions

Two types of soil were selected for this study in the north-west region of Colombia. The native soil was collected in a rural area near to the municipality of Angostura (Antioquia, 6° 53′ 7″ N, 75° 20′ 7″ W). This region has the same climatic conditions, altitude, and local plant diversity that have been reported for wild common bean populations collected in Colombia [23]. A successional forest was identified in the region, and soil samples were taken from the top layer (0–20 cm) after cleaning the litter, wood, and unwanted material. Several landraces typically associated with Mesoamerican traits were collected near this region; therefore, we refer to this soil as “native.” The agricultural soil was collected in a common bean producing farm in a rural area of the municipality of El Carmen de Viboral (6° 4′ 55″ N, 75° 20′ 3″ W). This soil was under cultivation for the last 30 years in a crop rotation system composed of maize, common bean, and potato. Tillage, liming, chemical fertilization (N-P-K), and the application of poultry waste are typical agricultural practices in the region. The climatic conditions, the altitude, and the local plant diversity in this region are not suitable for the growth of Colombian Mesoamerican wild common bean populations, but are optimal for Andean domesticated common bean varieties. Physicochemical analyses were performed in the Soil Science Laboratory from the National University of Colombia in Medellín, using standard procedures (Additional file 1: Table S1). Two wild, three landraces, and three improved varieties (cultivars) of common bean (*Phaseolus vulgaris*) were selected according to the following characteristics: belong to the Colombian Mesoamerican genetic pool, same race, similar phaseolin type, same altitudinal range, adapted to the same climatic conditions, and same growth type. The seeds were kindly provided by the Genetic Resources Program at the International Center for Tropical Agriculture - CIAT—in Palmira, Colombia. A genotypic analysis was performed on the common bean accessions to validate the domestication status provided by the original passport [10]. As a result, we reclassified the accessions as two wild (A1, A2), one landrace (L1), and five modern accessions (M1 to M5).

### Experimental design

Seeds of the eight bean accessions were surface-sterilized twice with sodium hypochlorite (0.5%) during 3 min and rinsed in sterile water four times. One hundred microliters of the last rinsing step was cultured in Tryptic Soy Agar (TSA, Oxoid) and in Potato Dextrose Agar (PDA, Difco) media by triplicate in order to check the growth of bacteria and fungi, respectively. Disinfected seeds were germinated on filter paper with sterile tap water; after 2 to 5 days, all the seeds had germinated. The native and agricultural soils were air-dried, passed through a 2-mm-mesh sieve, and distributed into 1 L PVC pots, with 700 g of dried soil per pot. Seedlings were transferred to the pots, with one plant per pot and four replicates per bean accession and per soil. The plants were cultivated in a growth chamber for 1 week and then arranged randomly in a screenhouse with an average temperature of 25 °C, 12 h of daylight, and daily watered with sterile tap water up to 70% of the maximum water holding capacity. Four pots with native soil and four pots with agricultural soil, both without plants were used as bulk soils.

### Sampling of rhizospheric soil

At flowering stage, the plants were carefully removed from the pots keeping the root system intact. Soil loosely adhered to the roots was removed by vigorous shaking, and when no more soil could be removed, the root system was submerged in tubes with 5 mL of LifeGuard Soil Preservation Solution (Mo Bio Laboratories, Carlsbad, CA, USA) and vigorously shaken in order to wash the roots and recover around 1 g of rhizospheric soil per sample for total DNA isolation. For the bulk soils, approximately 1 g of soil was collected from each control pot and also submerged in 5 mL of the LifeGuard solution. The LifeGuard Soil Preservation Solution can prevent microbial growth while maintaining nucleic acid integrity. All samples were kept at − 20 °C until further use.

### Total community DNA and RNA isolation

For each plant accession in each soil, four replicates of rhizospheric soil were used for total DNA extraction as well as four replicates of control soil. To obtain the total DNA, a two-step approach was followed in order to recover RNA and DNA from the same sample. First, RNA was isolated using the RNA PowerSoil Isolation Kit (Mo Bio Laboratories, Carlsbad, CA, USA) according to the manufacturer’s instructions, with slight modifications as follows. After adding the phenol:chloroform:isoamyl alcohol solution to the bead tube containing the bead solution, solution SR1 and SR2, as well as the soil sample, the agitation step was applied for 40 min. This modification allowed us to increase the RNA yields. The RNA was stored at − 80 °C for further use. For DNA isolation, the RNA PowerSoil® DNA Elution Accessory Kit (Mo Bio Laboratories, Carlsbad, CA, USA) was used. Briefly, after elution of the RNA from the RNA capture column, this column was transferred to another tube and the DNA elution procedure was performed according to the manufacturer’s instructions. Each obtained DNA sample was then cleaned with the PowerClean® DNA Clean-Up Kit (Mo Bio Laboratories, Carlsbad, CA, USA). Agarose gel electrophoresis and a ND1000 spectrophotometer (NanoDrop Technologies, Wilmington, DE, USA) were used to control RNA and DNA yield and quality. DNA samples were stored at − 80 °C until further use.

### 16S amplicon sequencing and bioinformatic processing

Total community DNA extracted from the rhizosphere was used for amplification and sequencing of the 16S rRNA, targeting the variable V3–V4 regions resulting in amplicons of approximately ~ 460 bp. Dual indices and Illumina sequencing adapters using the Nextera XT Index Kit were attached to the V3–V4 amplicons. Subsequently, library quantification, normalization, and pooling were performed and MiSeq v3 reagent kits were used to load the samples for MiSeq sequencing. For more info, please refer to the guidelines of Illumina MiSeq System [24]. The RDP extension to PANDASeq [25] named Assembler [26] was used to merge paired-end reads with a minimum overlap of 10 bp and at least a PHRED score of 25. Primer sequences were removed from the per sample FASTQ files using Flexbar version 2.5 [27]. Sequences were converted to FASTA format and concatenated into a single file. All reads were clustered into OTUs using the UPARSE strategy by dereplication, sorting by abundance with at least two sequences and clustering using the UCLUST smallmem algorithm [28]. These steps were performed with VSEARCH version 1.0.10 [29], which is an open-source and 64-bit multithreaded compatible alternative to USEARCH. Next, chimeric sequences were detected using the UCHIME algorithm [30] implemented in VSEARCH. All reads before the dereplication step were mapped to OTUs using the usearch_global method implemented in VSEARCH to create an OTU table and converted to BIOM-Format 1.3.1 [31]. Finally, taxonomic information for each OTU was added to the BIOM file by using the RDP Classifier version 2.10 [26]. All steps were implemented in a Snakemake workflow [32].

### Diversity and abundance analysis

For downstream analysis, we took the obtained OTU table and prepared a “filtered table” using QIIME (1.9.1) custom scripts [33]. First, we extracted from the OTU table the bacteria domain using the command *split_otu_table_by_taxonomy.py*. Next, we discarded singletons, doubletons, chloroplast, and mitochondria sequences using the command *filter_otus_from_otu_table.py*. With the “filtered_OTUtable,” we first calculated the alpha diversity. Using the command *alpha_rarefaction.py*, the OTU table was rarefied to counts up to 35,000 reads. The reason to use this value was because this was the lowest sequencing depth obtained from a sample. To calculate the diversity indexes, we used the *alpha_diversity.py* and *alpha_rarefaction* commands, obtaining Shannon, observed OTUs, Chao1, and Faith’s Phylogenetic Diversity metrics. One-way ANOVA and Tukey HSD, as well as statistical tests to validate ANOVA assumptions were performed in R (3.4.1) [34]. For the beta diversity, the unrarefied “filtered_OTUtable” was first normalized using the R package metagenomeSeq (v.1.12) [35]. We used a cumulative-sum scaling (CSS) method to avoid the biases generated with current sequencing technologies due to uneven sequencing depth [36]. With the normalized OTU table Bray-Curtis, weighted and unweighted Unifrac dissimilarity matrices were calculated and used to perform Principal Coordinate Analysis with Phyloseq package (v.1.10) [37]. The nonparametric *adonis* test was used to assess the percentage of variation explained by the soil type along with its statistical significance using Vegan (v.2.4-0) package [38], all performed in R. For the differential abundance analysis and the construction of the heat maps, the STAMP software (v.2.1.3) was used [39]. Rarefied OTU tables from Agricultural and Native rhizosphere and bulk soil data (35,000 reads per sample) were used for pairwise comparisons. Welch’s t tests followed by Bonferroni corrections were performed at phylum and at family levels between soils. Dendograms were built in STAMP with an average neighbor method (UPGMA), and the rows included all the bacterial phyla observed in rhizosphere and bulk soil samples along with their relative abundance.

In order to compare the number of shared and exclusive rhizobacterial genera between common bean accessions in agricultural and native soil, we selected wild accessions A1 and A2 and modern bean accessions M3 and M4. These four accessions were selected in order to normalize the number of samples as well as the number of reads to compare. Regarding the modern accessions, M3 and M4 showed to be the most modern accessions available both for native and agricultural soils [10]. To depict the taxa exclusively found in a particular soil or accession, we used the online tool Venny (2.1) [40], and to graphically represent the exclusive genera, we built Euler diagrams using the shiny app eulerr of the homonymous R package [41]. Euler diagrams are area-proportional generalized Venn diagrams for which the requirement that all intersections be present is relaxed. Euler diagrams were built for the exclusive and shared genera per soil type and plant domestication status.

In order to have a better understanding of the composition of the bacterial diversity in the rhizosphere of the common bean accessions, we calculated several species abundance distribution models and determined whether neutral or niche-based mechanisms were governing the bacterial assembly. We hypothesized that the agricultural soil would be driven by neutral-based processes and that the native soil would respond to niche-based process. We used the command Radfit from the R package vegan to evaluate several abundance models and a zero-sum multinomial (ZSM) model. Species abundance distributions models and comparison of the models fit based on the Akaike Information Criterion (AIC) were calculated as previously reported [10].

### Core microbiome and co-occurrence network analyses

For the core microbiome analyses, rarefied OTU tables (35,000 reads each sample) were used for both soils. The QIIME command *compute_core_microbiome.py* was applied in order to obtain a list of OTUs observed in 100% of the common bean rhizosphere samples regardless of soil type. Core microbiome analyses were also performed for common bean on each soil type. Only core OTUs with a relative abundance > 0.5% were included for graphical purposes. Pie and donut charts were built in R. Network analysis was performed to assess the complexity of the interactions among microbial taxa in the common bean rhizosphere grown in the agricultural soil (*n* = 32) and in the native soil (*n* = 26). Best practices for co-occurrence network construction were strictly followed [42]. Rarefied OTU tables were filtered to a minimum threshold of 25 sequences per OTU. Non-random co-occurrence analyses were performed using SparCC [43]. *P* values were obtained by 99 permutations of random selections of the data table. SparCC correlations with a magnitude > 0.8 or ≤ 0.8 and statistically significant (*P* < 0.01) were further included into network construction. The nodes in the reconstructed networks represent the OTUs at 97% identity, whereas the edges correspond to a strong and significant correlation between nodes. The topology of the network was inferred on a set of measures (number of nodes and edges, modularity, number of communities, average path length, network diameter, averaged degree, and clustering coefficient) which were calculated using Gephi (v.0.9.2) [44]. Network visualizations were constructed using Cytoscape (v. 3.4.0) [45]. Clusters were calculated using a hierarchical clustering algorithm (HC-PIN) with the Cytoscape plugin Cytocluster [46].

## Results

### Soil and genotype influence plant growth and development

Eight accessions of common bean, encompassing wild relatives, landraces, and modern cultivars were grown in agricultural and native soils collected from the Colombian highlands, at the same time under the same screenhouse conditions (see the “Material and methods” section). The soils differed in several physicochemical characteristics (Table 1). Briefly, the agricultural soil had more organic matter, available phosphorus, and calcium as well as higher pH and cation exchange capacity (CEC) as compared to the native soil. The native soil showed higher concentrations of iron and aluminum. At flowering stage, each bean accession was harvested to collect rhizospheric soil and to assess several plant phenotypic traits. In the agricultural soil, significant differences were observed between the bean accessions in root dry weight and the number of days to reach flowering stage (Table 2). Genotype-dependent differences were also observed in the native soil (Table 2). Several replicates of the accession M5 did not grow in the native soil or showed a poor development and were therefore not included in further analyses. In general, the mean root dry weight was higher for bean accessions grown in the agricultural soil than in the native soil (Additional file 1: Figure S1a). Finally, the number of days to reach the flowering stage (R6) was higher in the native soil than in the agricultural soil (Additional file 1: Figure S1b).

**Table 1:** Soil physical and chemical analyses. Physical and chemical characterization of the agricultural and the native soil used in this study to grow the common bean plants for the rhizobacterial characterization

Unit	Item		Agricultural	Native
%	Texture	Clay	8	24
		Silt	30	12
		Sand	62	64
		Classification	Clay loam	Sandy clay loam
dSm^−1^	pH		5.8	4.7
%	Organic matter		17.9	11.6
cmolc kg^−1^	Al		ND	3.00
	Ca		15.0	0.3
	Mg		2.9	1.0
	K		0.6	0.4
	Na		0.03	0.03
	CEC		18.50	4.80
mg kg^−1^	P		56	3
	S		8	8
	Fe		50	620
	Mn		2	2
	Cu		3	3
	Zn		7	4
	B		0.24	0.21

Methods: Texture: Bouyoucos; pH: water (1:1); organic matter: Walkley Black; Al: KCl 1M; Ca, Mg, K, Na: ammonium acetate 1M; CEC: cation exchange sum; S: monocalcium phosphate 0.008M; Fe, Mn, Cu, Zn: Olsen-EDTA; B: hot water; P: Bray II. Unit consideration: *ND* not detectable, dSm^−1^ = mmho cm^−1^, cmolc kg^−1^ = meq/100 g soil, mg kg^−1^ = ppm

**Table 2:** Phenological traits of the common bean accessions grown in native and agricultural soils. Data for root dry weight and the number of days to reach the flowering stage is shown

	Root dry weight (g)		Days to flower	
Accession	Native	Agricultural	Native	Agricultural
A1	0.082 ± 0.015 bcd	0.438 ± 0.141 ab	111.0 ± 12.3 a	93.5 ± 12.4 a
A2	0.139 ± 0.010 a	0.484 ± 0.149 a	103.7 ± 11.4 ab	83.5 ± 5.7 ab
L1	0.118 ± 0.009 ab	0.282 ± 0.080 bc	81.0 ± 31.0 bc	62.5 ± 10.8 c
M1	0.112 ± 0.056 abc	0.219 ± 0.053 c	90.3 ± 17.0 abc	64.8 ± 16.7 bc
M2	0.134 ± 0.039 a	0.374 ± 0.215 abc	75.0 ± 4.0 c	66.5 ± 21.2 bc
M3	0.055 ± 0.016 d	0.260 ± 0.127 c	87.6 ± 11.4 abc	64.8 ± 16.7 bc
M4	0.067 ± 0.019 cd	0.204 ± 0.046 c	106.6 ± 3.5 ab	56 ± 5.2 c
M5	ND*	0.332 ± 0.048 abc	ND	56 ± 5.2 c

The mean values of four replicates (agricultural) and three replicates (native) per accession are shown, followed by the standard deviation of the mean. The harsh conditions of the native soil prevented us to have four replicates for all the accessions; therefore, it was decided to normalize the number of replicates to three in native soil samples. Statistical analysis of root dry weight and days to flowering were done between bean accessions per soil type. ANOVA and LSD (*P* < 0.05) tests were applied after checking for assumptions of normality and homoscedasticity. Accessions with the same letter are not significantly different

*Accession M5 did not grow on the native soil

### Diversity of rhizobacterial communities is driven by soil type and rhizosphere

**Fig. 1:**
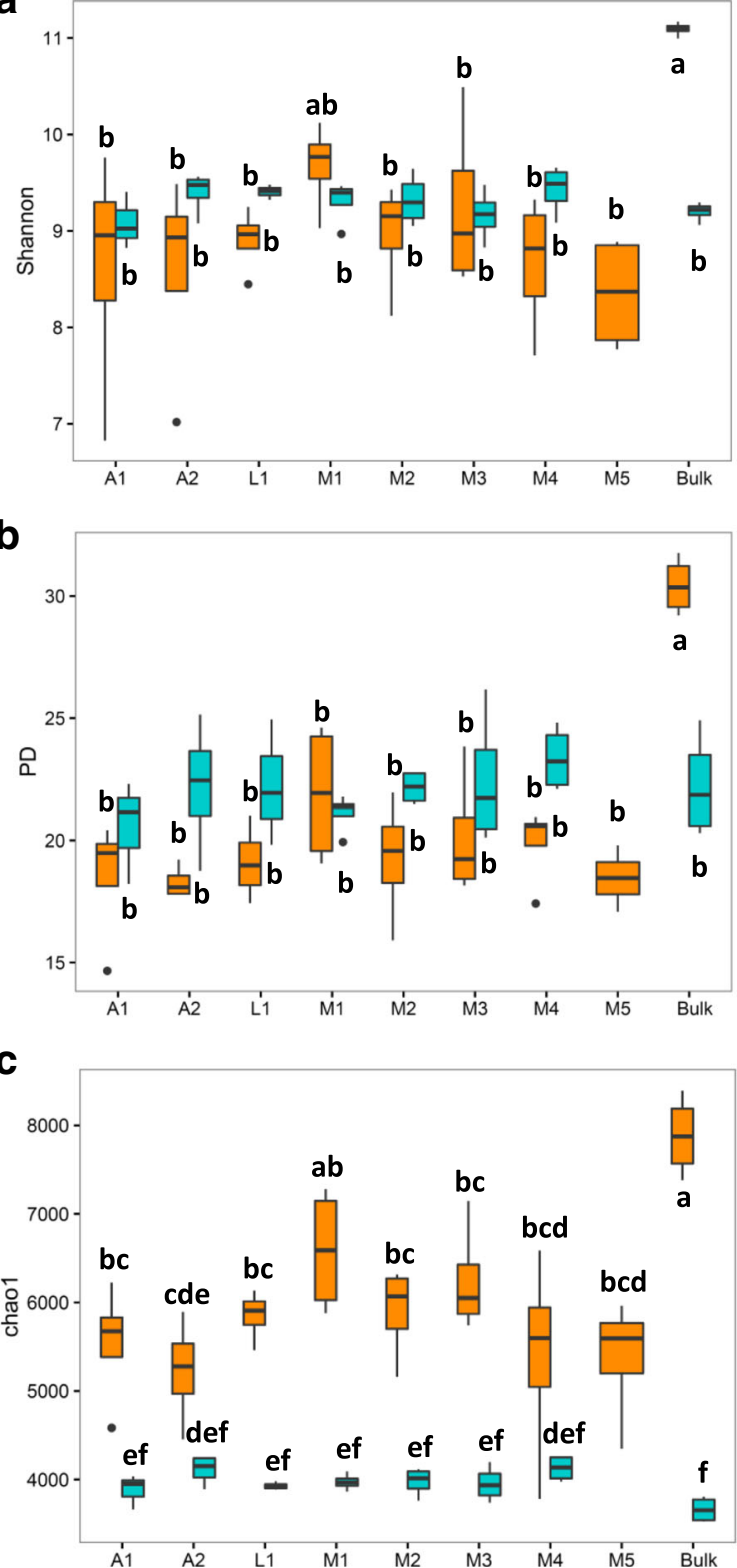
Comparative analysis of the alpha diversity of 16S rRNA rhizobacterial sequences from common bean accessions in agricultural and native soils. **a** Shannon, **b** phylogenetic diversity, and **c** Chao1 were calculated by soil type and for all bean accessions and the bulk soils. The data was rarefied up to 35,000 counts per sample. Statistically significant differences were determined by one-way ANOVA (*P <* 0.05) followed by post hoc Tukey test. Cyan color was assigned to native soil and dark orange to agricultural samples

**Fig. 2:**
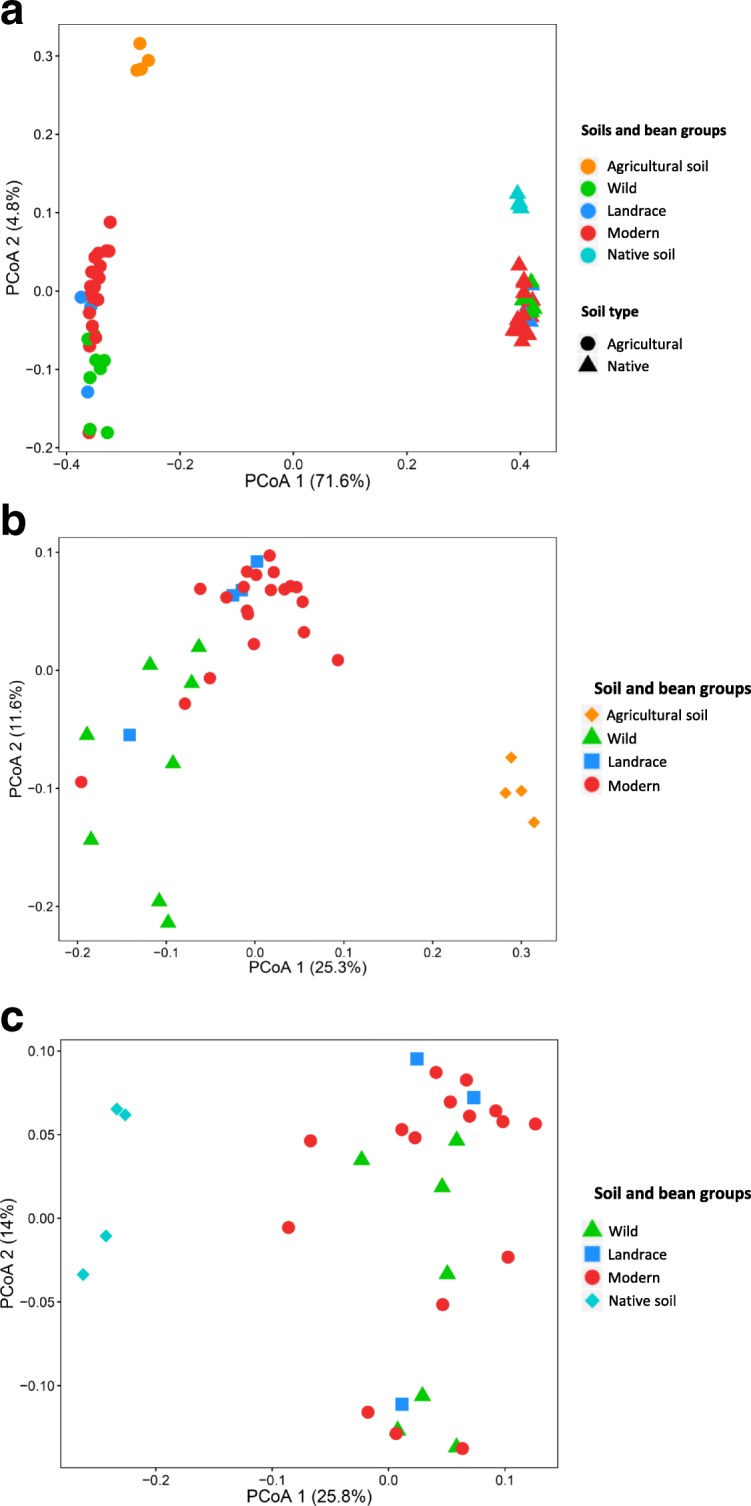
Rhizosphere bacterial community structure in agricultural and native soils. Principal Coordinate Analysis (PCoA) of 16S rRNA diversity in the rhizosphere of the eight common bean accessions used in this study. **a** Rhizosphere bacterial community of common bean grown in agricultural (circles) and native (triangles) soils. Soil type explained 71.3% of the total variability in the bacterial community composition (PERMANOVA, *P <* 0.001). **b** PCoA including only rhizosphere bacterial communities of common bean plants grown in agricultural rhizosphere and bulk soil samples. Bean genotype explained 31.2% of the total variability in the agricultural soil (PERMANOVA, *P* < 0.05). **c** PCoA including only rhizosphere bacterial communities of bean plants grown in native rhizosphere and bulk soil samples. Bean genotype explained 28.3% of the total variability in the agricultural soil (PERMANOVA, *P* < 0.001). CSS transformed reads were used to calculate Bray-Curtis distances in **a**, **b**, and **c**. Colors represent the stage of domestication and bacterial communities from agricultural and native bulk soils

For bulk soil and rhizosphere samples, 4.24 million reads were recovered after quality filtering (Additional file 1: Table S1), representing 16.727 bacterial operational taxonomic units (OTUs) at 97% sequence similarity. For the *α*-diversity, rarefaction curves were obtained for Chao1, observed OTUs, and phylogenetic diversity (PD) indices (Additional file 1: Figure S2). The evenness, represented by the Shannon index and the phylogenetic diversity (PD), was in general similar between rhizosphere samples in both soil types (Fig. 1a and b), while the bacterial species richness was significantly higher in the agricultural soil than in the native soil (Fig. 1c). In the agricultural soil, all diversity indices were significantly higher for the bulk soil than for the rhizosphere samples. Additionally, it was observed that agricultural bulk soil samples showed significantly higher values for all the diversity indices as compared to the native bulk soil samples. Regarding the *β*-diversity, Bray-Curtis metrics and Principal Coordinate Analysis (PCoA) revealed a significant effect of the soil type (Fig. 2a). Soil type alone explained 71.3% of the total variability in the bacterial community composition (PERMANOVA, *P <* 0.001). Subsequently, the samples were divided by soil type and analyzed separately. For the total variability of the rhizobacterial community structure, the bean genotype explained 31.2% in the agricultural soil (PERMANOVA, *P* < 0.001) and 28.3% in the native soil (PERMANOVA, *P* < 0.05) (Fig. 2b and c). Unifrac metrics confirmed the results observed with Bray-Curtis metrics (Additional file 1: Figure S3). The mean Bray-Curtis distances showed that the variability of the rhizobacterial communities within samples of the same accession was significantly lower as compared to the distance between the bean accessions (agricultural soil, *P <* 0.001; native soil, *P <* 0.05; *t* test, Bonferroni-corrected). For the agricultural soil, the rhizobacterial community composition of wild bean accession A1 was similar to that of wild accession A2, but significantly different from that of the landrace and the five modern bean accessions (*P <* 0.001; *t* test, Bonferroni-corrected). In the native soil, however, rhizobacterial community composition did not differ significantly between the wild and modern bean accessions.

### Specific differences in microbiome between native and agricultural soils

**Fig. 3:**
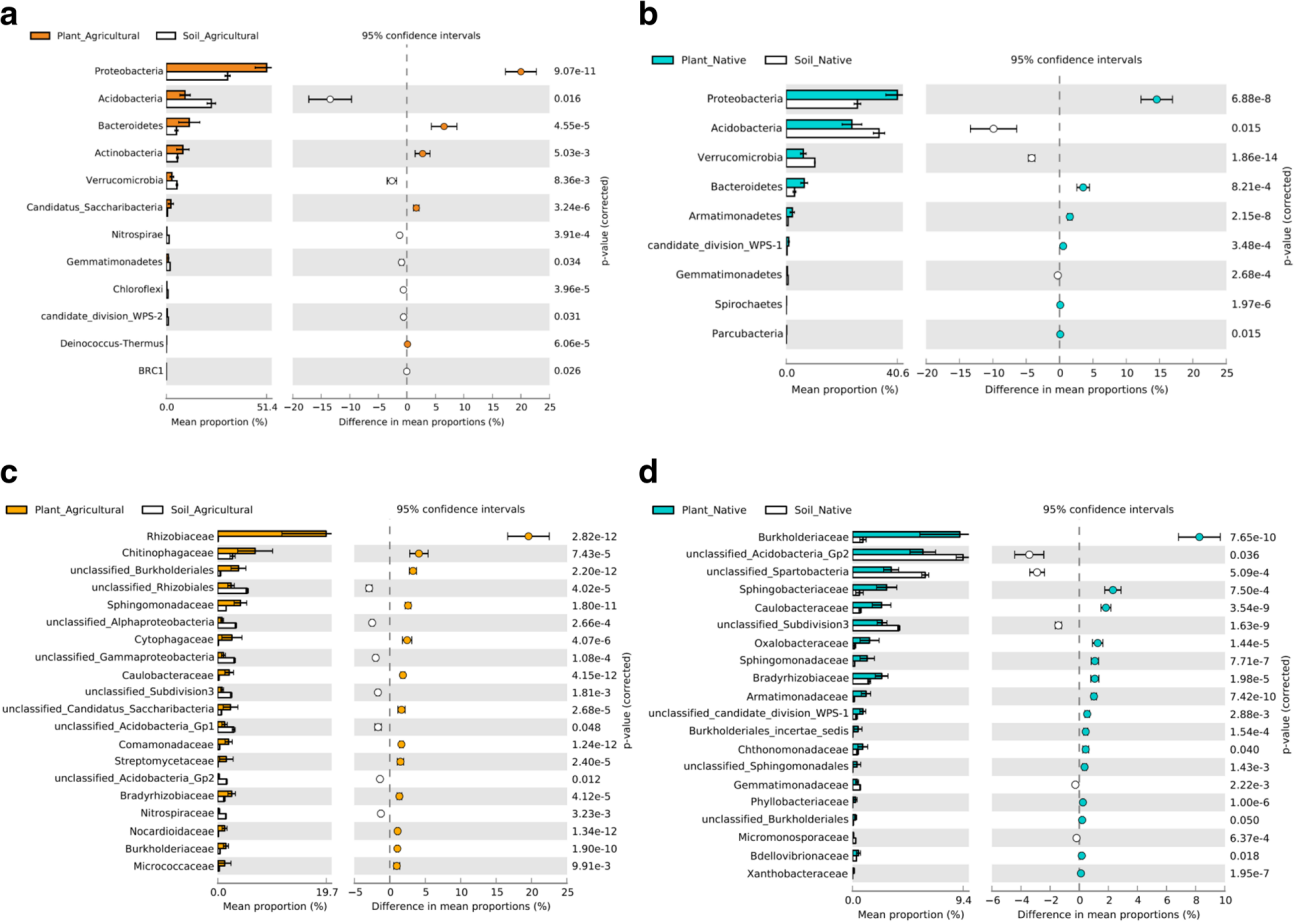
Differential abundance of bacterial OTUs in agricultural and native soils. Welch’s *t* tests followed by Bonferroni corrections were performed between merged rhizosphere samples and merged bulk soil samples from agricultural soil and native soil at phylum (**a** and **c**) and class (**b** and **d**) levels. Only differentially abundant phyla and classes are shown

The observed differences in *α-* and *β*-diversity between the native and agricultural soils and between the eight bean accessions led us to explore more in-depth the differences in taxonomic identity and relative abundance of the bacterial taxa for each soil. The most abundant bacterial phyla were *Proteobacteria*, *Acidobacteria*, and *Bacteroidetes* in both soils. In the native soil, however, the phylum *Acidobacteria* showed a higher relative abundance than in the agricultural soil (Additional file 1: Figures S4 and S5). At phylum level, *Acidobacteria* and *Verrucomicrobia* were significantly more abundant in the native bulk soil than in the agricultural bulk soil (Welch’s *t* test, *P <* 0.05, Bonferroni-corrected) (Additional file 1: Figure S6a). At class level, *Acidobacteria* subgroups 1, 2, and 3 were enriched in the native soil, while *Acidobacteria* subgroup 4 and *Betaproteobacteria* were more abundant in the agricultural soil (Welch’s *t* test, *P <* 0.05, Bonferroni-corrected) (Additional file 1: Figure S6b). *Proteobacteria* and *Bacteroidetes* were consistently more abundant in the rhizosphere of common bean, regardless of the soil type, while *Acidobacteria* and *Verrucomicrobia* showed a consistent decrease in the rhizosphere compared to their abundance in bulk soil (Fig. 3a and b). *Actinobacteria* was significantly more abundant in the rhizosphere of common bean grown in the agricultural soil than in the bulk agricultural soil (Fig. 3a), whereas this rhizosphere effect was not observed in the native soil (Fig. 3b) (Welch’s *t* test, *P <* 0.05, Bonferroni-corrected). Among the *Actinobacteria* enriched in the rhizosphere of all bean accessions grown in the agricultural soil, *Streptomycetaceae* and *Nocardioidaceae* were the most abundant families together with *Rhizobiaceae*, *Sphingomonadaceae*, *Caulobacteraceae*, and *Comamonadaceae* for the *Proteobacteria* and *Chitinophagaceae* and *Cytophagaceae* for the *Bacteroidetes* (Fig. 3c). The smaller yet significant rhizosphere effect observed for the eight bean accessions grown in the native soil was explained by higher relative abundances of *Burkholderiaceae*, *Caulobacteraceae*, *Oxalobacteraceae*, *Sphingomonadaceae*, and *Bradyrhizobiaceae* for the *Proteobacteria* and *Sphingobacteriaceae* for the *Bacteroidetes* (Fig. 3d). To further dissect these differences in microbiome composition between rhizosphere and bulk soils, the abundance of the read counts was fitted to several species abundance distribution (SAD) models. Comparison of Akaike’s Information Criterion (AIC) allowed us to find the best-fit value from six distribution models. The results showed that the OTU abundance distributions in the rhizosphere of the bean accessions grown in agricultural and native soils, and the respective bulk soils, are explained by niche-based distributions [8] (Additional file 1: Table S2).

### Higher rhizobacterial diversity for common bean in agricultural soil

We performed a comparison of the bean rhizobacterial community at genus level between soil types and between wild and modern bean accessions in order to decipher if/how habitat expansion and agricultural soil management may have depleted or enriched rhizosphere bacterial diversity. The results showed that 143 rhizobacterial genera, representing 28.7% of the total number of genera, were exclusively represented in the agricultural soil (Additional file 1: Figure S7a). Exclusive genera accounted for 2.3% of the total relative abundance in agricultural soil. Some of these “exclusive” genera such as *Lysobacter* and *Aeromicrobium* accounted for 0.6% and 0.4% of the total relative abundance, respectively. Thirty-one genera including *Cytophaga* and *Acidicapsa* were exclusively found in the native soil, representing 6.2% of the total number of rhizobacterial genera (Additional file 1: Figure S7a). Exclusive genera accounted for 0.2% of the total relative abundance in native soil.

Two wild bean accessions (A1 and A2) and two modern accessions (M3 and M4) were selected for further analysis to compare the number of shared and exclusive bacterial genera in the rhizosphere. We found that in the agricultural soil, 85.9% of the rhizobacterial genera were shared between wild and modern accessions, 8.7% was exclusively found in the rhizosphere of the modern bean accessions, and 5.4% was exclusively found in the rhizosphere of the wild accessions (Additional file 1: Figure S7b). In the native soil, a similar trend was observed, with 84.8% of the rhizobacterial genera shared between wild and modern bean accessions, 9.0% exclusively found in the rhizosphere of the two modern accessions, and 6.3% in the two wild accessions (Additional file 1: Figure S7c). In conclusion, we found more bacterial genera in the rhizosphere of the eight bean accessions grown in the agricultural soil than in the native soil. Additionally, we found more bacterial genera in the rhizosphere of the modern bean accessions than in wild accessions irrespective of the soil type. It should be noted that the abundance of these “exclusive” bacterial genera in the common bean rhizosphere was relatively low for both soils.

### The core microbiome of common bean is represented by a small subset of rhizobacterial genera

**Fig. 4:**
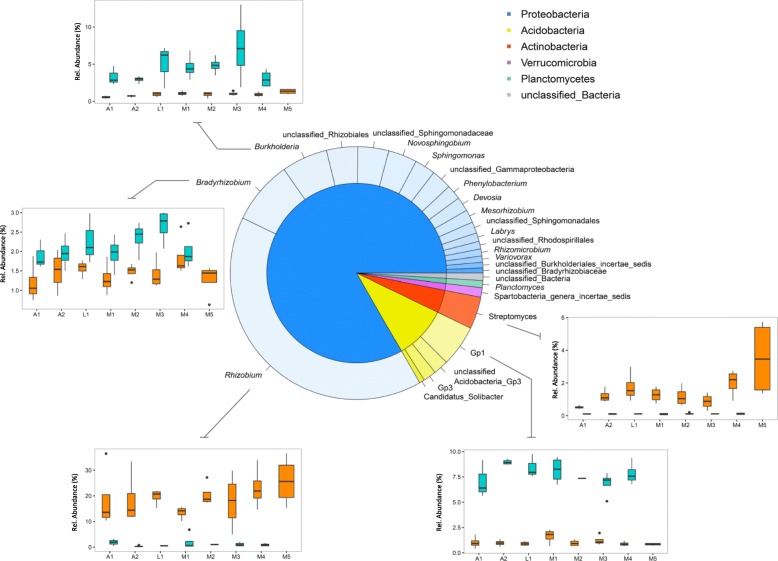
Core microbiome of the rhizosphere of common bean. The different portions within the inner pie chart represent the bacterial phyla that are part of the common bean core microbiome. The outer donut plot represents the genera that are part of the core, and each genus assigned to the phylum they belong to. The size of the different pie and donut portions represents the contribution of each phylum/genus to the total relative abundance. Satellite box plots depict the relative abundance of selected genera by bean accession (A1 and A2, wild; L1, landrace; M1 to M5, modern) and by soil type. Cyan and dark orange colors were assigned to native soil and agricultural samples, respectively

From the total of 16,727 clustered OTUs, we found 113 OTUs consistently present in the rhizosphere of all eight bean accessions grown in the native and agricultural soils. These 113 OTUs, classified up to genus level, represented only 0.67% of the total number of OTUs but 25.9% of all the sequence reads. This core bean rhizosphere microbiome consisted of 61 *Proteobacteria* OTUs that made up 68.8% of the mean relative abundance with the genus *Rhizobium* as the most abundant contributor (two OTUs, 33.4%), followed by *Bradyrhizobium* (two OTUs, 6.7%), *Burkholderia* (three OTUs, 4.9%), *Novosphingobium* (three OTUs, 3.0%) and *Sphingomonas* (one OTU, 2.2%) (Fig. 4). Other phyla represented in the core rhizosphere microbiome were *Acidobacteria* (27 OTUs, 12.2% relative abundance), *Actinobacteria* (six OTUs, 4.1%), *Verrucomicrobia* (eight OTUs, 2.5%), and *Planctomycetes* (five OTUs, 1.1%). A core microbiome analysis was done also per soil type in order to dissect the specific contribution of each habitat to the overall core. For the agricultural soil, the core rhizobacterial microbiome was composed of 611 OTUs representing 4.97% of the total number of OTUs and 33.07% of the reads. *Proteobacteria* (219 OTUs), *Bacteroidetes* (62 OTUs), and *Actinobacteria* (58 OTUs) were the three most abundant phyla within the core (Additional file 1: Figure S8) with again *Rhizobium* as the most abundant genus (26.7%) followed by *Dyadobacter* (3.3%) and *Streptomyces* (2.1%). In the native soil, the core rhizosphere microbiome was composed of 812 OTUs representing 12.6% of the total number of OTUs and 46.4% of the reads. *Proteobacteria* (237 OTUs), *Acidobacteria* (190 OTUs), *Verrucomicrobia* (68 OTUs), *Bacteroidetes* (53 OTUs), *Actinobacteria* (48 OTUs), and *Chloroflexi* (17 OTUs) were the most abundant phyla (Additional file 1: Figure S9). Within *Proteobacteria*, *Ralstonia* was the most abundant genus (4.6%) followed by *Burkholderia* (4.0%), *Herbaspirillum* (1.6%), and *Rhizobium* (1.2%). In the core rhizosphere microbiome of beans grown in the native soil, *Acidobacteria* was mainly represented by the *Acidobacteria* subgroups 1, 2, and 3, with 27.4% of the reads. In fact, less than 3% of the OTUs classified in the core as *Acidobacteria* summed up 12.7% of the total number of reads, evidencing the strong dominance of this phylum in the native soil habitat. *Verrucomicrobia* represented in total 6.6% of the core microbiome with most of the reads assigned as *incerta sedis*. Finally, the genus *Mucilaginibacter* and unclassified members of the *Chitinophagaceae* family accounted for most of the abundance of *Bacteroidetes* representing 5.4% of the core; for *Actinobacteria*, small contributions mostly by unclassified *Acidimicrobiales* and *Actinomycetales* collectively accounted for a relative abundance of 3.1%. In conclusion, these comparative analyses indicated that only a small number of 113 bacterial OTUs were consistently present in the rhizosphere of all eight bean accessions grown in the agricultural and native soils and also revealed that these OTUs represent on average more than a quarter (25.9%) of the total of 4.2 million sequence reads.

### Higher co-occurrence network complexity in native soil

**Fig. 5:**
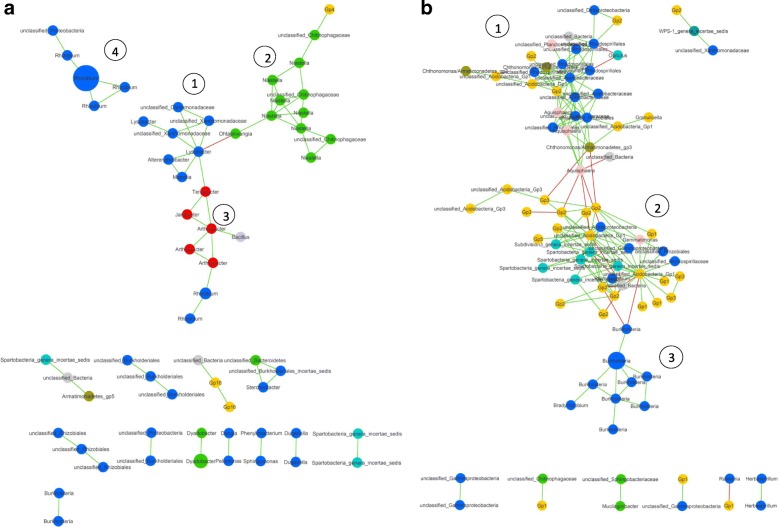
Common bean rhizobacterial co-occurrence networks in agricultural and native soils. **a** Co-occurrence network of common bean rhizosphere samples in agricultural soil. Cluster 1 was composed of bacterial taxa from several classes of the *Proteobacteria* phylum. Cluster 2 contained exclusively bacterial taxa from the *Chitinophagaceae* family. Cluster 3 included actinobacterial taxa and one *Bacillus*, and cluster 4 was composed of the genus *Rhizobium*. **b** Co-occurrence network of common bean rhizosphere samples in native soil. From the three main clusters identified, two were highly abundant in nodes from the *Proteobacteria* phylum (1 and 3) which held negative connections to cluster 2, mainly composed of phyla *Acidobacteria* and *Verrucomicrobia*. Positive interactions are depicted as green edges and the negative interactions are depicted as red edges. Color code of most abundant nodes: Proteobacteria, blue; Actinobacteria, red; Bacteroidetes, green; Acidobacteria, yellow; Planctomycetes, cyan

Co-occurrence network analyses were performed to assess the complexity of the interactions between the rhizobacterial taxa detected in the rhizosphere of common bean grown in native and agricultural soils. The correlations between the occurrence of the rhizobacterial genera were calculated using SparCC [43] followed by the graphical inference of the network and the estimation of several topological properties (Additional file 1: Table S3). The rhizobacterial network in agricultural soil consisted of 63 nodes and 61 significant correlations, with only one negative connection between OTUs identified as *Lysobacter* and *Ohtaekwangia* (Fig. 5a). In general, this network presented a simple structure, with four main clusters and few OTUs per cluster. For the native soil, the obtained network contained 89 nodes and 176 significant correlations, with 158 positive and 18 negative (Fig. 5b). Three main clusters were identified, with a high number of nodes per cluster and with a high number of interconnections within each cluster. Remarkably, cluster 2 was connected to the other two clusters only through negative correlations. Global network statistics were determined for both networks (Additional file 1: Table S3). Briefly, modularity and the number of communities were higher in the agricultural soil than in the native soil. Conversely, the average path length and the average degree were higher in the native soil. Using Betweenness Centrality (BC), we aimed to find keystone species within each network. In the agricultural soil, the highest BC values were found for the genera *Lysobacter* (OTU_136), *Rhizobium* (OTU_1), *Niastella* (OTU_10281, OTU_44, and OTU_56), *Ohtaekwangia* (OTU_69), *Terrabacter* (OTU_46), and *Arthrobacter* (OTU_886). For the native soil, the highest BC values were found for *Aquisphaera* (OTU_537, *Planctomycetes* phylum), two unclassified *Acidobacteria* (OTU_62 and OTU_12725), and an unclassified *Acetobacteraceae* (OTU_175) and *Burkholderia* (OTU_45).

## Discussion

In this study, we showed that rhizobacterial diversity of wild and domesticated common bean (*Phaseolus vulgaris*) was higher in an agricultural soil than in a native soil. Furthermore, species abundance analyses revealed niche-based processes for both soils suggesting selection pressures. For the agricultural soil, management practices (fertilization, addition of organic matter) are the most likely drivers of the observed differences in species abundance distributions, whereas edaphic factors, in particular low pH, are the most probable selection pressures for the native soil. Bacterial diversity is generally lower in acidic soils [47, 48], and pH largely determines the composition of the soil bacterial communities [49]. Our results further showed that the impact of the bean genotype on rhizobacterial assembly was more prominent in the agricultural soil than in the native soil where the rhizosphere effect was much smaller and where genotype-dependent effects on rhizobacterial community composition were more homogeneous. An underlying mechanism of this minor and more homogenous rhizosphere effect is that the harsh abiotic conditions in the native soil may have affected the amount and quality of root exudates released into the soil. In the native soil used in this study, the bean plants faced a low soil pH, high aluminum concentrations, and low *P* availability, characteristics that are common for tropical undisturbed soils [15, 50]. Also, the lack of nodulation in these acidic conditions [51] could have undermined symbiotic associations for nitrogen uptake and concomitantly the growth and development of the common bean plants with an adverse effect on root exudation.

Common bean grown in the agricultural soil harbored more exclusive OTUs than bean grown in the native soil, and we also found more exclusive OTUs in the rhizosphere of modern bean accessions as compared to wild accessions, irrespective of the soil type. The genera exclusive for the agricultural soil were *Lysobacter* and *Aeromicrobium*. The genus *Lysobacter* is commonly found in agricultural soils [52], and their abundance is strongly modulated by soil type and negatively affected by low pH [53, 54]. Liming is a common agricultural practice in tropical croplands to increase soil pH [55] and is also typically applied in the region in Colombia where the agricultural soil used in our study was collected. Consequently, their exclusive presence in the agricultural soil might be related with the higher pH as compared to the acidic native soil. Also, the exclusive genus *Aeromicrobium* prefers neutral to alkaline pH and has been previously isolated from agricultural fields [56, 57]. In terms of activity, both *Lysobacter* and *Aeromicrobium* species are known to produce diverse secondary metabolites, with antimicrobial properties [58–61] which may aid in the protection of the bean plants against biotic stress caused by soil-borne pathogens. Further experimentation is needed to decipher the impact of these “enriched” microbes for growth and health of common bean in agricultural and native habitats.

Bacteria exclusively found in the native habitat of wild relatives of crop plants comprise representatives of *Cytophaga* and *Acidicapsa* genera. The genus *Cytophaga* is known for its cellulose-degrading capabilities, and species such as *Cytophaga hutchinsonii* can be found as indigenous soil inhabitants [62, 63]. Their exclusive presence in the native soil may be associated with their ability to decompose complex carbohydrates such as plant litter and decaying wood, thereby contributing to carbon cycling in the undisturbed native soils. The genus *Acidicapsa*, which belongs to the phylum *Acidobacteria*, encompasses strictly aerobic chemo-organotrophs that are adapted to acidic conditions [64, 65]. *Acidobacteria* members are in general considered oligotrophs and have been found positively associated with low soil pH [66, 67]. The diversity and abundance of acidobacterial species in soil, as well as their diversity in metabolic traits, makes *Acidobacteria* a potentially important phylum in soil nutrient cycling [68, 69]. If these rhizobacterial genera, when re-introduced into agricultural soils, will be able to establish and survive in the rhizosphere of modern bean cultivars and, if they can, provide additional life-support functions (growth, health) for the bean plants remains to be investigated. It is important to highlight that the enriched or depleted bacterial taxa explored in this study are based on amplicon sequences that were classified up to genus level. It is possible that bacterial species that were classified up to genus level in our analysis are absent in one of the soil types. Therefore, additional analyses that allow taxonomic resolution at the species or preferably at the strain level are needed.

The members of the core microbiome shared by all eight bean accessions in both soils were in general very abundant. The core microbiome genera included *Rhizobium*, *Bradyrhizobium*, *Mesorhizobium*, *Sphingomonas*, and *Streptomyces*. These results showed that a significant portion of the core microbiome of common bean is composed of bacterial genera with nitrogen-fixing capabilities, an important feature of microbes associated with leguminous plant species. However, also for other non-leguminous plant species, these rhizobacterial genera are members of the core rhizosphere microbiome [70–72]*.* We further observed that *Rhizobium* was by far the most dominant core member in the agricultural soil while in the native soil the genera *Burkholderia*, *Ralstonia*, and unclassified *Rhizobiales* were the dominant core members. These latter genera are most likely better adapted to acidic conditions in the native soil and probably responded more efficiently to root signals, such as flavonoids released by roots of common bean. *Burkholderia* species are indeed well represented in acidic soils [73] and have been found as nodule-forming rhizobia in symbiosis with leguminous plants [74–76] including common bean [77]. To form nodules, however, compatibility between *Burkholderia* spp. and the legume host is a key factor [76]. In fact, common bean nodulation in tropical acid soils in South America has been associated with only a few *Rhizobium* species [78, 79] which were found in low abundances in the native soil. Despite the high abundance of *Ralstonia* in the native soil, a genus known to harbor soil-borne bacterial pathogens, no disease symptoms in common bean roots were evidenced in our study. *Ralstonia* species may occupy several ecological niches and have been isolated from different environments, including soil [80]. It has been shown that tropical leguminous plants can be nodulated by *Ralstonia taiwanensis* that display functional nitrogenase activity [81]. Nevertheless, whether the *Burkholderia* and *Ralstonia* OTUs detected here in the rhizosphere can establish symbiotic associations with common bean is not known yet.

The co-occurrence network analyses further indicated that the interactions between rhizobacterial taxa in the rhizosphere of common bean accessions grown in a native soil environment were more complex than those in an agriculturally managed soil, where the establishment of copiotrophs in the rhizosphere compartment was favored. Based on these results, we hypothesize that rhizobacterial community assembly for common bean grown in agricultural soil is less complex and more modular than for common bean in native soil. This in turn makes it relatively more easy for a given soil bacterial species to invade and establish in the rhizosphere of bean plants grown in the agricultural soil. Following this hypothesis, the higher rhizobacterial diversity observed for common bean in the agricultural soil may represent a less specialized microbiome. Along these lines, previous studies have indicated that N-fertilization of soil induces shifts in bacterial community composition, promoting copiotrophs that rely on labile carbon sources [13], and promotes the evolution of less mutualistic microbes [12]. In the “agricultural” and “native soil” networks, we observed positive interactions between nodes, which suggest niche overlap, as well as negative interactions, suggesting competition or amensalism [82]. The occurrence of phylogenetically related OTUs was in general positively correlated, forming well-differentiated clusters (Fig. 5). Accordingly, it has been shown that *Acidobacteria* and *Verrucomicrobia* phyla co-occur more than expected by chance only [83]. In this study, we found a similar pattern in the native soil network, where cluster 2 is composed mainly of the oligotrophic phyla *Acidobacteria* and *Verrucomicrobia*. This cluster interacts negatively with clusters 1 and 3, abundant in copiotrophic bacterial genera, that presumably respond better to the common bean root exudates. Furthermore, the clustering suggests a strong niche differentiation [82]. For instance, cluster 4 in the agricultural network is composed exclusively of rhizobial OTUs, with no interactions with other clusters. Similarly, cluster 2 consisted mainly of *Bacteroidetes* that may represent the rhizobacterial hub involved in degradation of complex polymers [84, 85]. Also, cluster 3 in the native soil, mainly composed of *Burkholderia*, might represent a specific hub of nodule-forming rhizobia [77]. Whether these hubs represent distinct functional groups remains to be investigated by metagenomics and trait-based bioassays.

It is well stablished that plant domestication and subsequent improvement of crop cultivars caused phenotypic, genomic, and metabolic changes which enabled the use of plants by humans [86–88]. Many of these changes were accompanied by other inadvertent effects, such as the reduction in the genetic diversity of domesticated crop cultivars [2], and the negative impact of the domesticated crops to deal with herbivorous insects [89]. Regarding the effect of plant domestication on the rhizosphere microbiome, divergences in the structure of the microbial communities associated with wild and cultivated plant species have been repeatedly found. Similarly, it has been observed that the abundance of certain taxa was reduced or augmented in domesticated/wild plants. Studies conducted with sugar beet [7], barley [8], and lettuce [90] showed an enrichment of members of the *Bacteroidetes* in the rhizosphere of the wild relatives as compared to their domesticated counterparts [6]. Similarly, the bacterial genera *Flavobacterium* and *Pedobacter*, both from the *Bacteroidetes* phylum, were enriched in wild rice as compared to cultivated rice [91]. For common bean, we previously showed that wild accessions grown in agricultural soil were enriched with families within the *Bacteroidetes* phylum as compared to modern accessions [10]. This specific enrichment of the phylum *Bacteroidetes* in the rhizosphere of wild accessions was not observed in the native soil and might be related with the harsh abiotic conditions of this soil as discussed above. Despite the studies conducted so far have revealed differences on the abundance of particular rhizosphere microbial taxa between domesticated crops and wild counterparts, the mechanisms, genotypic and phenotypic traits, or chemical interactions behind these changes are still unknown as well as its consequences on plant health and development.

## Conclusions

Our study showed that the transition of common bean from a native soil to an agricultural soil led to a gain of rhizobacterial diversity. We found a low diverse but highly abundant core microbiome which resembles that of other plant species, suggesting a homogenization of rhizobacterial diversity of plants grown in different agricultural landscapes. It is important to note that the core microbiome analysis presented here is based solely on taxonomy and that functional traits should be taken into account in future analyses for better insight into the impact of habitat expansion on trait-based microbiome assembly [92]. The network structure was simpler in agricultural soil as compared to native soil, which again may reflect the process of biotic homogenization. In this study, we also aimed for the identification of microbes that were depleted as a consequence of domestication and habitat expansion of common bean. Indeed, several bacterial genera were exclusively found in the native soil and also as an exclusive member of the rhizosphere of wild bean accessions. These bacterial genera were low-abundant members of the rhizobacterial community. Conversely, the number of bacterial taxa exclusively found in the agricultural soil was considerably higher. The proportion of depleted bacterial genera appears to be overcompensated in the agricultural soil by the number of “gained microbes,” many of which were highly abundant in the rhizosphere of all eight common bean accessions. On the other hand, this increased bacterial diversity in the agricultural soil might also correspond to a less specialized microbiome. To what extent these “enriched” and “depleted” bacterial genera have an impact on plant growth and health is not yet known and subject of future experiments. It is important to emphasize that the number of agricultural and native soils tested should be further expanded to resolve if the significant changes we observed between the two soils tested in our study can be extrapolated as general trends in rhizobacterial shifts during domestication.

## Additional file

Additional file 1: **Figure S1.** Phenological traits of the common bean accessions grown in native and agricultural soils. **Figure S2.** Rarefaction curves for Chao1, observed OTUs and phylogenetic diversity metrics. **Figure S3. **Rhizosphere bacterial community structure using weighted Unifrac metrics in agricultural and native soils. **Figure S4.** Relative abundance of the most abundant bacterial phyla in agricultural and native soils. **Figure S5. **Heat map of the relative abundance of all the bacterial phyla in agricultural and native soils. **Figure S6.** Differential abundance of bacterial phyla and classes between bulk soil samples of the agricultural soil and the native soil. **Figure S7.** The enriched and depleted bacteria in the rhizosphere of wild and modern common bean grown in native and agricultural soils.** Figure S8.** Core microbiome in the rhizosphere of common bean grown in agricultural soil. **Figure S9.** Core microbiome in the rhizosphere of common bean grown in native soil. **Table S1.** Sequence characteristics obtained in the agricultural soil and native soil. **Table S2.** AIC values for six rank abundance distribution models to test niche neutral models in native soil samples. **Table S3.** Co-occurrence network properties of 16S rRNA rhizobacterial reads of common bean rhizosphere in agricultural and native soil. (PDF 1910 kb)

## Data Availability

The datasets generated and analyzed during the current study are available in the European Nucleotide Archive (ENA) repository under the accession number PRJEB26084.
