# Supplementary material for: Deciphering rhizosphere microbiome assembly of wild and modern common bean (Phaseolus vulgaris) in native and agricultural soils from Colombia
Source: Microbiome. 2019 Aug 14;7:114. doi: 10.1186/s40168-019-0727-1 (PMC6694607; doi:10.1186/s40168-019-0727-1)
Supplement: Supplementary file 1 — Figure S1. Phenological traits of the common bean accessions grown in native and agricultural soils. Figure S2. Rarefaction curves for Chao1, observed OTUs and phylogenetic diversity metrics. Figure S3. Rhizosphere bacterial community structure using weighted Unifrac metrics in agricultural and native soils. Figure S4. Relative abundance of the most abundant bacterial phyla in agricultural and native soils. Figure S5. Heat map of the relative abundance of all the bacterial phyla in agricultural and native soils. Figure S6. Differential abundance of bacterial phyla and classes between bulk soil samples of the agricultural soil and the native soil. Figure S7. The enriched and depleted bacteria in the rhizosphere of wild and modern common bean grown in native and agricultural soils. Figure S8. Core microbiome in the rhizosphere of common bean grown in agricultural soil. Figure S9. Core microbiome in the rhizosphere of common bean grown in native soil. Table S1. Sequence characteristics obtained in the agricultural soil and native soil. Table S2. AIC values for six rank abundance distribution models to test niche neutral models in native soil samples. Table S3. Co-occurrence network properties of 16S rRNA rhizobacterial reads of common bean rhizosphere in agricultural and native soil. (PDF 1910 kb) [file 40168_2019_727_MOESM1_ESM.pdf]

Additional file 1

Deciphering rhizosphere microbiome assembly of wild and modern common bean (Phaseolus vulgaris) in native and agricultural soils from Colombia

Juan E. Pérez-Jaramillo[1,2], Mattias de Hollander[1], Camilo A. Ramírez[3], Rodrigo Mendes[4], Jos M. Raaijmakers[1,2*] and Victor J. Carrión[1]

Affiliations:
[1]Department of Microbial Ecology, Netherlands Institute of Ecology (NIOO-KNAW), P.O. Box 50, 6708 PB Wageningen, The Netherlands.
[2]Institute of Biology, Leiden University, Sylviusweg 72, 2333 BE, Leiden, The Netherlands.
[3]Institute of Biology, University of Antioquia, Calle 67 #53-108, Medellín, Colombia.
[4]Embrapa Meio Ambiente, Rodovia SP 340 - km 127.5, 13820-000 Jaguariúna, Brazil.

*Corresponding Author
Prof. dr. Jos M. Raaijmakers.
Netherlands Institute of Ecology (NIOO-KNAW)
Department of Microbial Ecology
P.O. Box 50, 6708 PB
Wageningen, The Netherlands.
Telephone number: +31 (0)317 473 497.
e-mail: J.Raaijmakers@nioo.knaw.nl

Supplementary figures

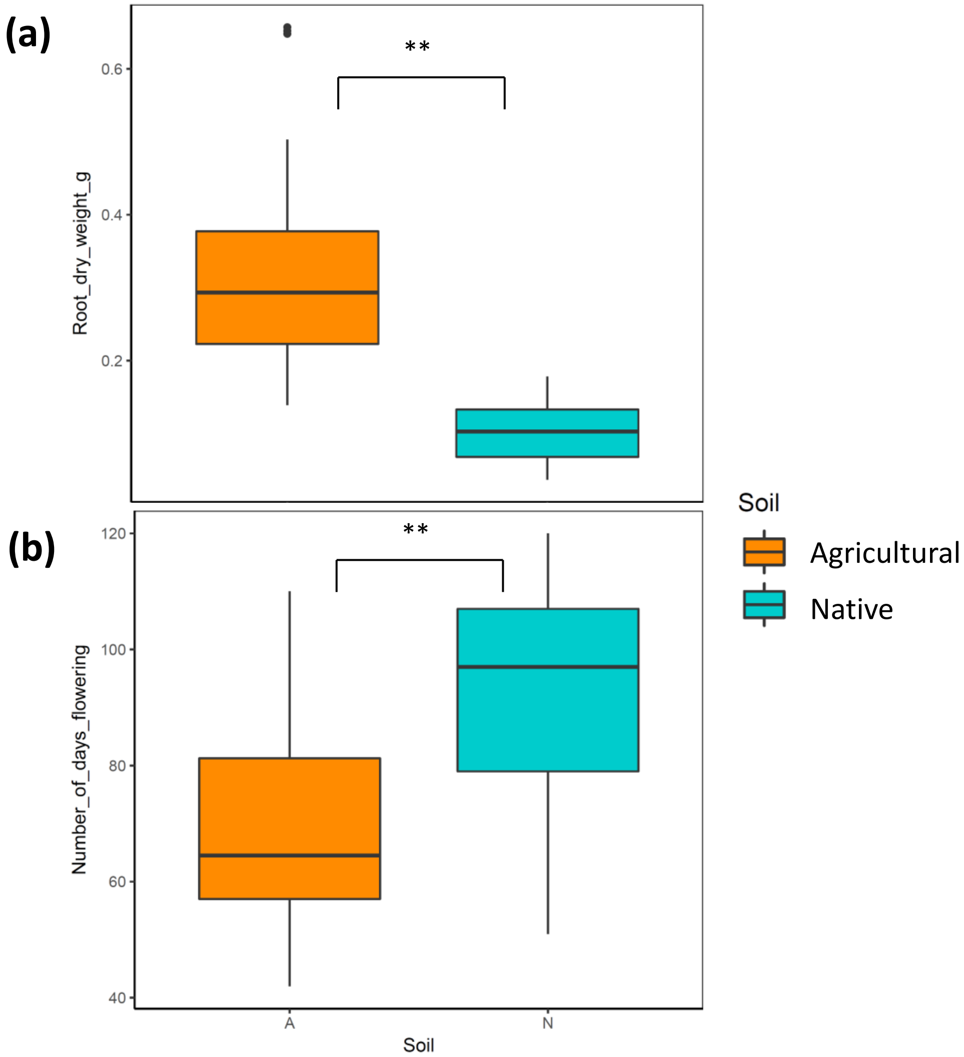

Figure S1. Phenological traits of the common bean accessions grown in native and agricultural soils. Data for root dry weight (a) and the number of days to reach the flowering stage (b) of common bean accessions grown in agricultural and native soils was merged per soil type and used to built box plots. Subsequently, kruskal and Wallis test was applied followed by a Bonferroni correction. (** significantly different with a P<0.05)

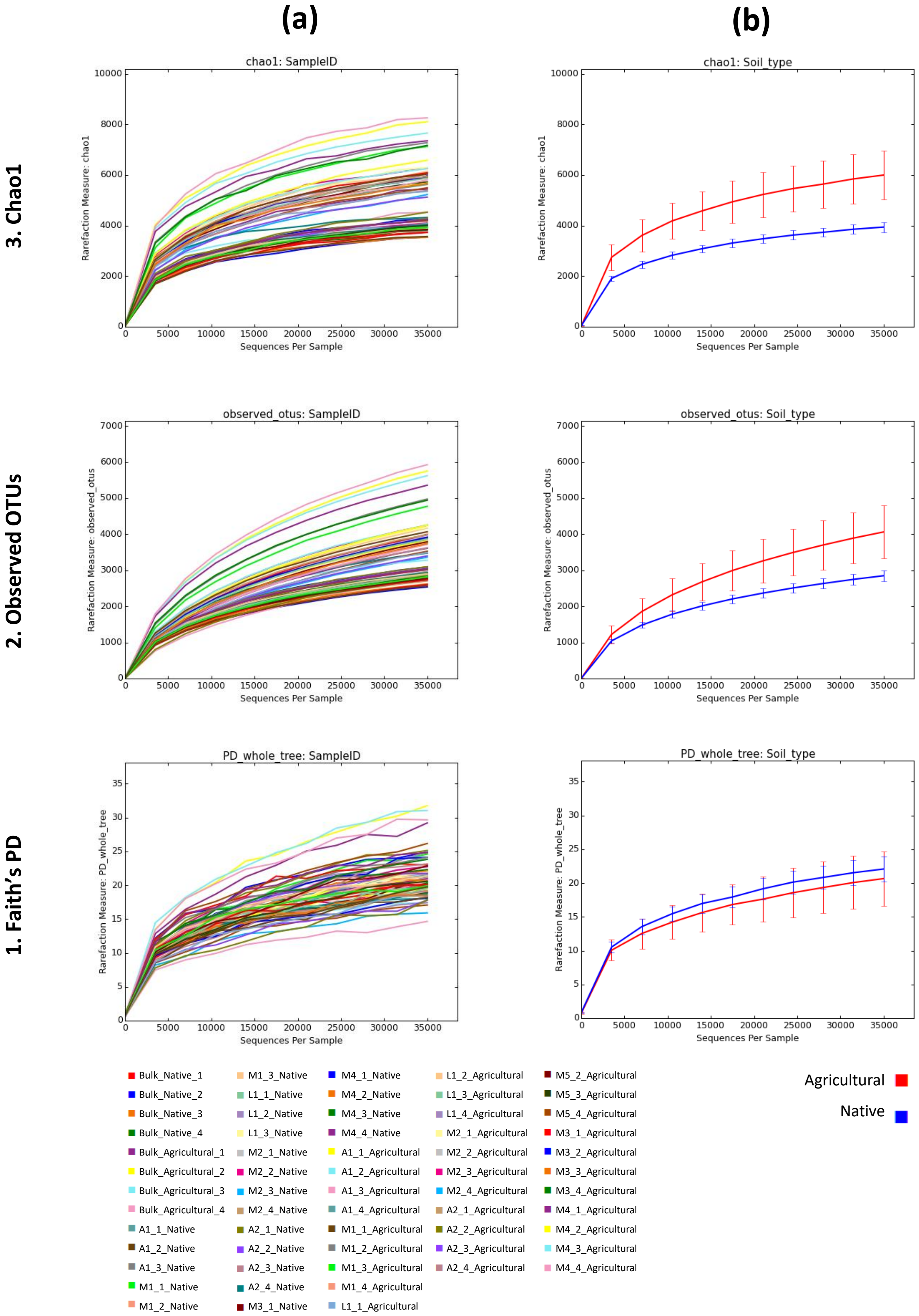

Figure S2. Rarefactions curves for chao1, observed OTUs and phylogenetic diversity. metrics. In the rows, the rarefaction curves are shown by index as follows: 1. Faith's phylogenetic diversity; 2. Number of observed OTUs; 3. chao1 index for estimated OTUs. In the rows, rarefaction curves are shown by (a) Sample; (b) Soil type.

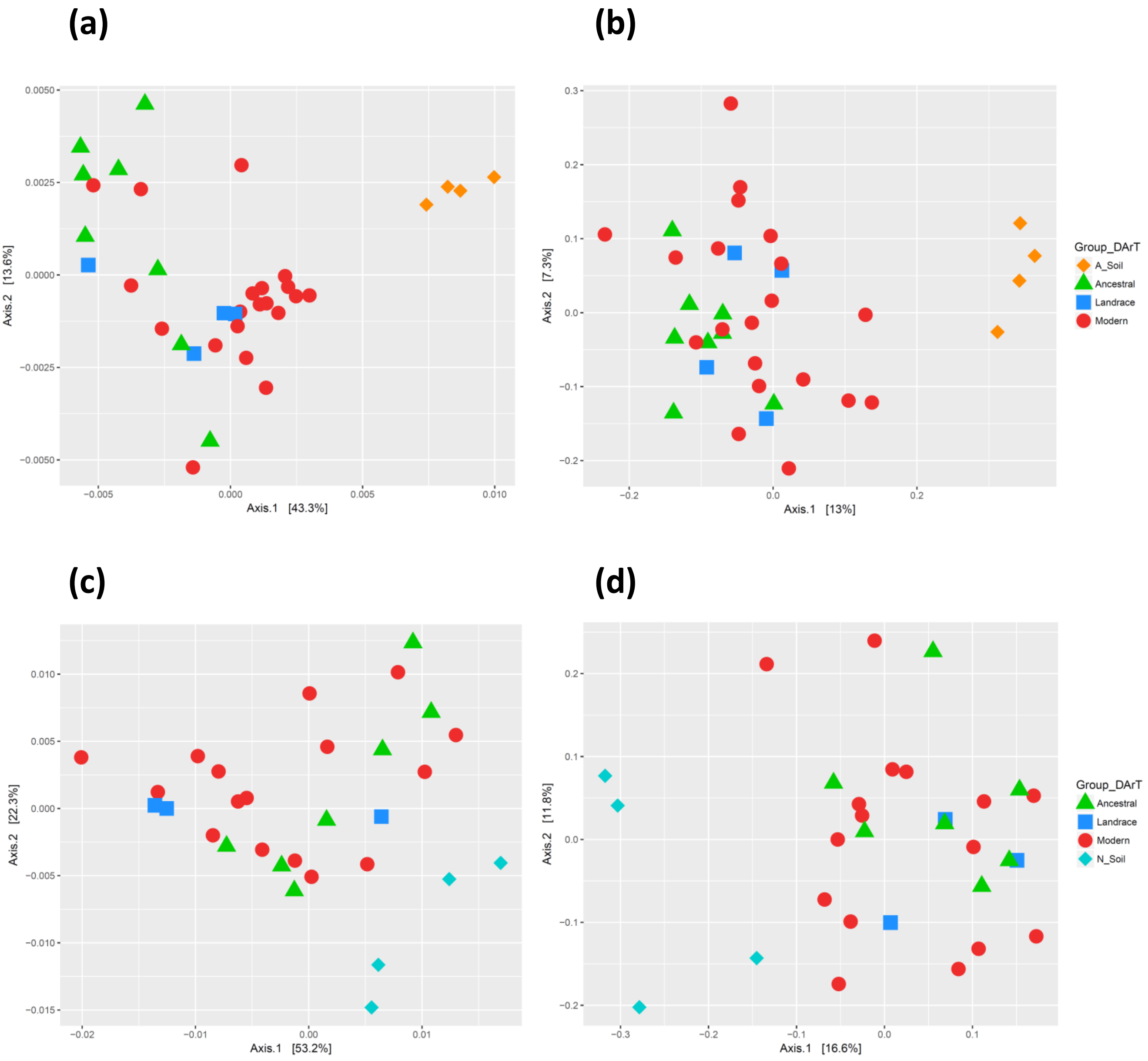

Figure S3. Rhizosphere bacterial community structure using weighted Unifrac metrics in agricultural and native soils. Principal Coordinate Analysis (PCoA) of 16S rRNA diversity in the rhizosphere of the eight common bean accessions used in this study. (a) weighted and (b) unweighted unifrac metrics of agricultural rhizosphere and soil samples and (c) weighted and (d) unweighted unifrac metrics of native rhizosphere and soil samples.

(a)

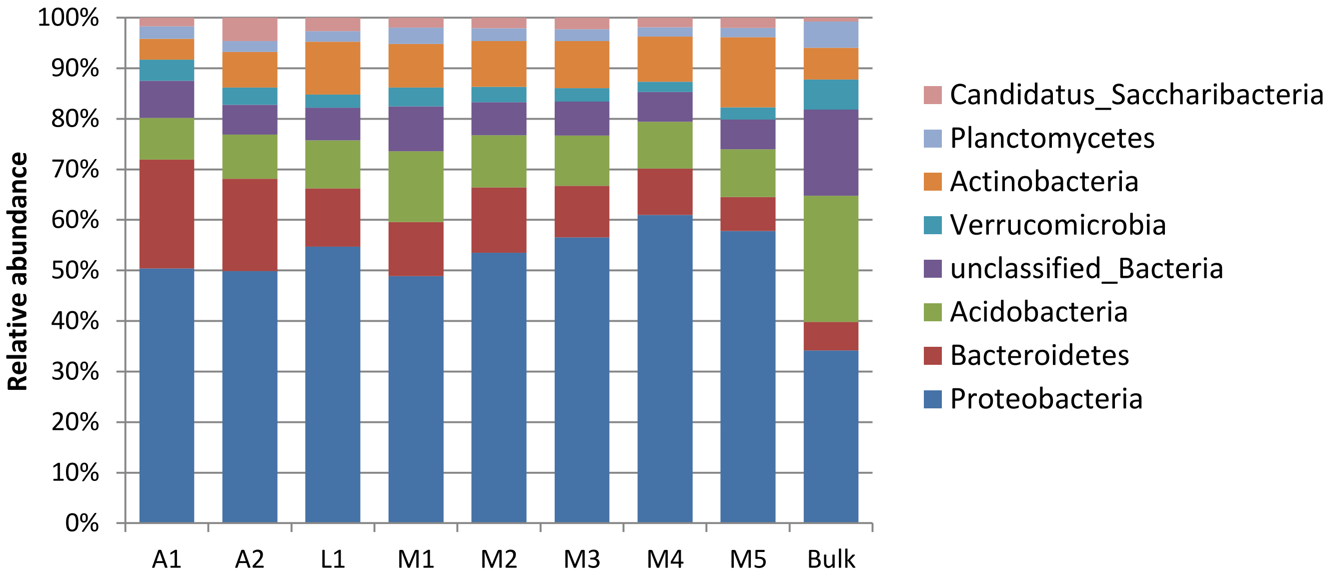

(b)

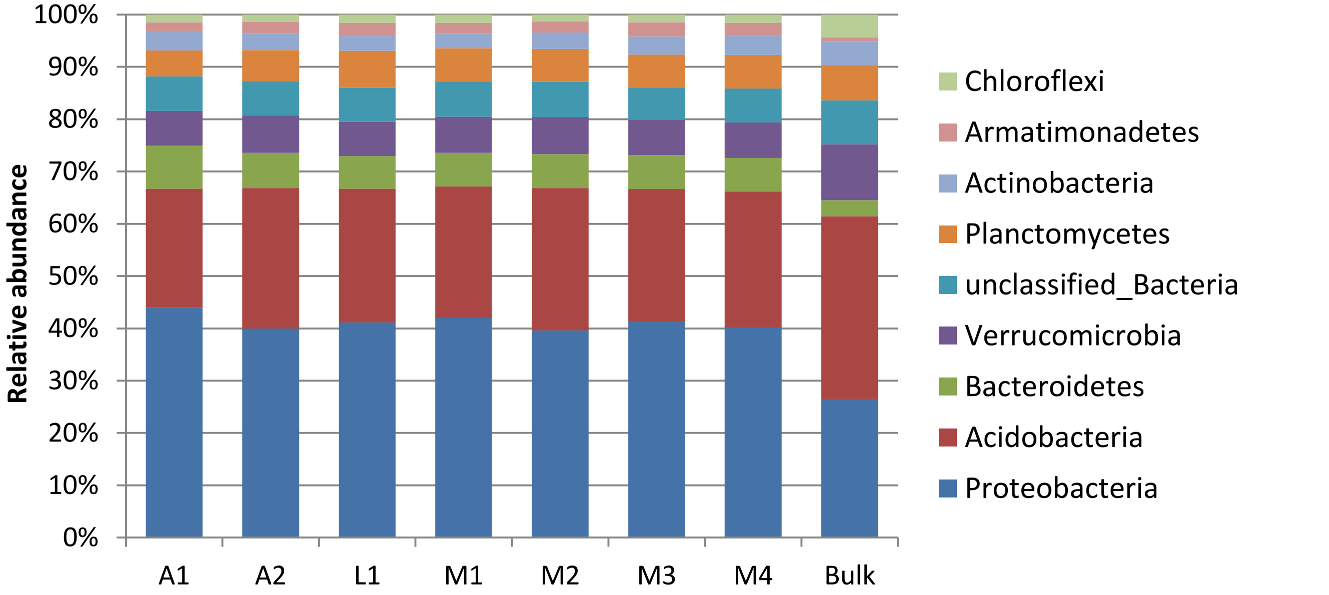

Figure S4. Relative abundance of the most abundant bacterial phyla in agricultural and native soils. Bar graphs of the relative abundance of the most abundant bacterial phyla in the agricultural (a) and in the native (b) soil are shown. Only phyla with a total relative abundance higher than 1% are depicted in the graphs.

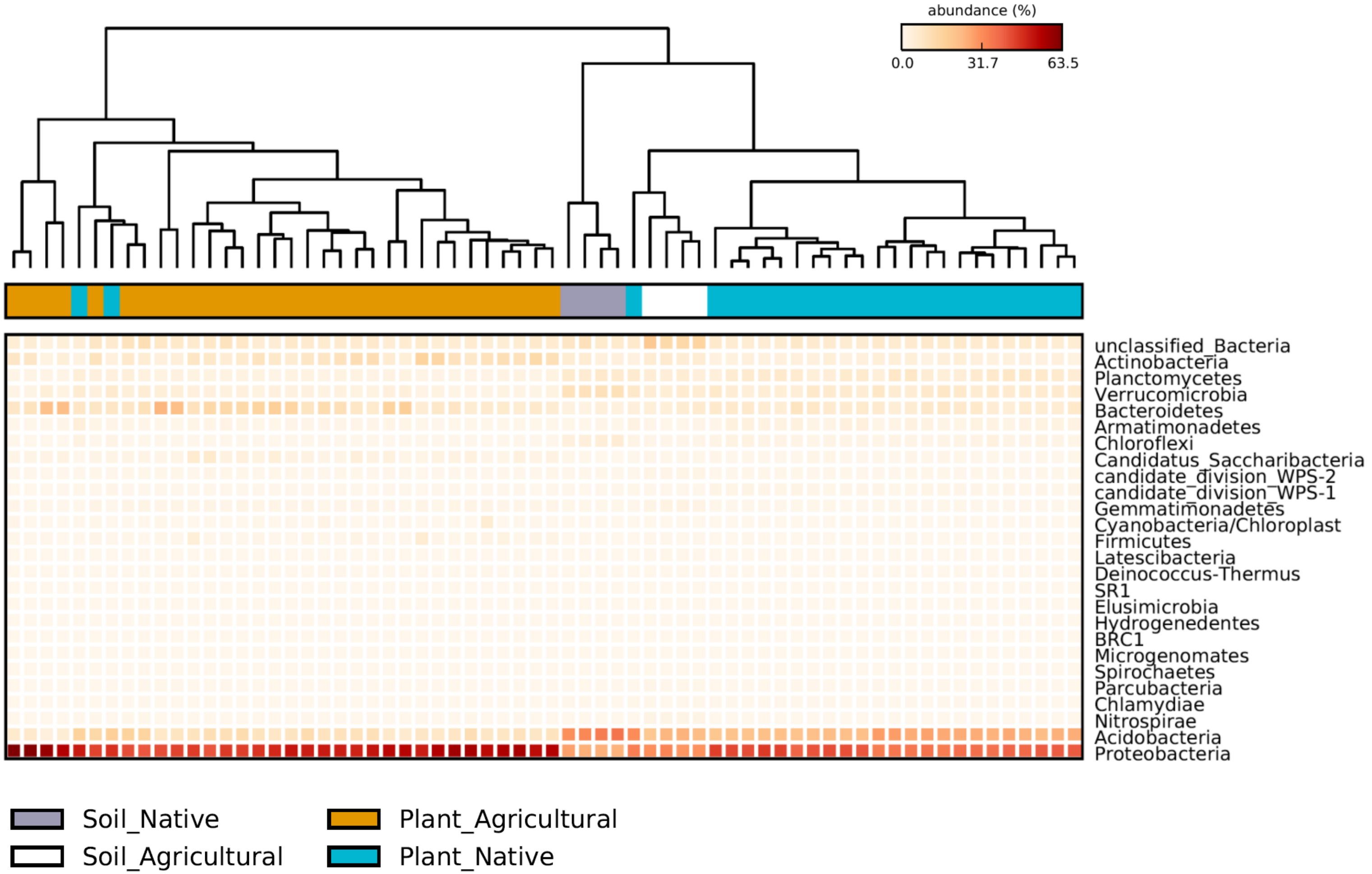

Figure S5. Heat map of the relative abundance of all the bacterial phyla in agricultural and native soils. The columns indicate the common bean accessions clustered through a dendogram built with an average neighbor method (UPGMA) and the rows shows the bacterial phyla observed in rhizosphere and bulk soil samples. The darker the red squares in the heat map the more abundant the phylum of the specific row. Color codes for the samples are indicated in the lower section of the graph.

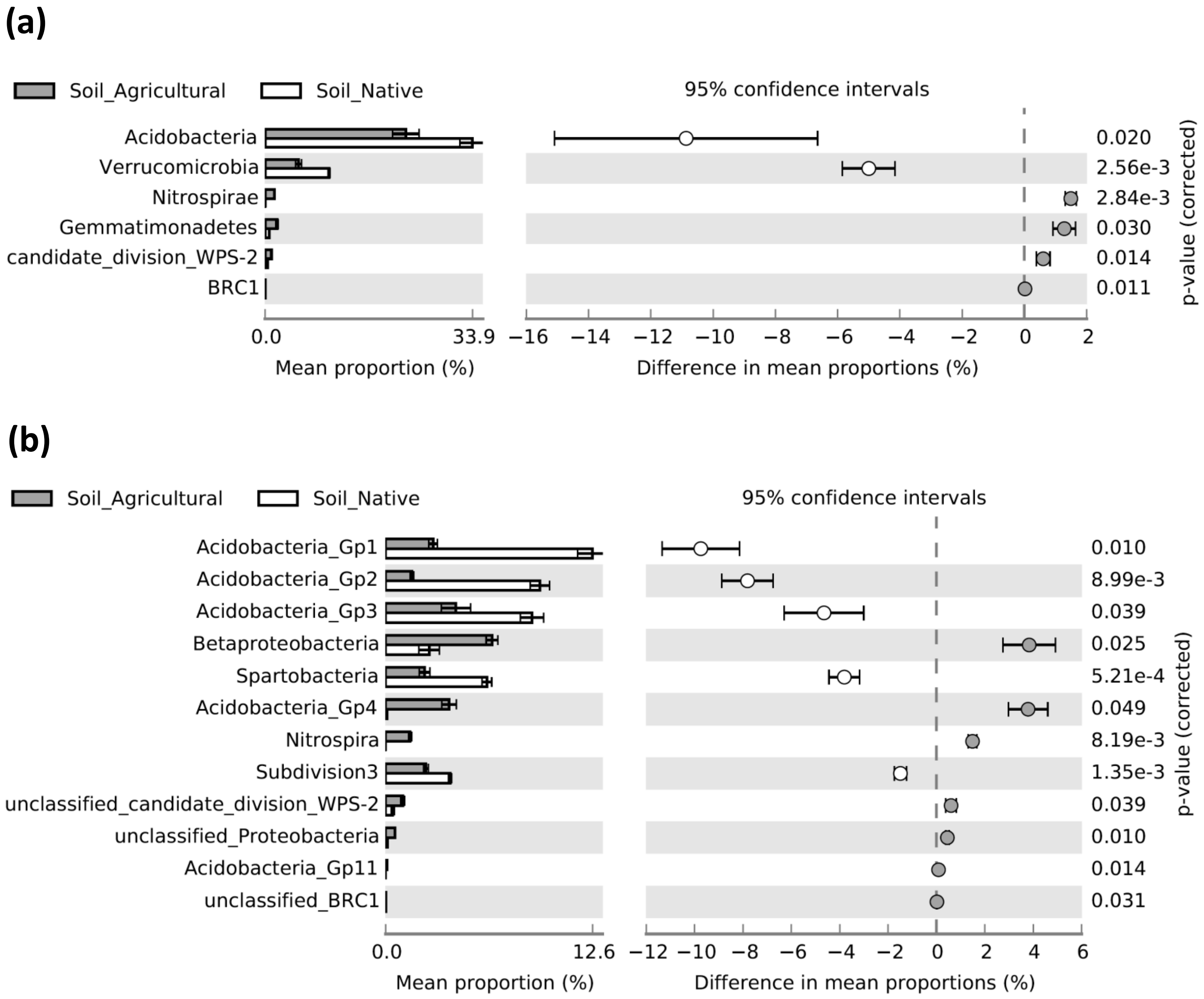

Figure S6. Differential abundance of bacterial phyla and classes between bulk soil samples of the agricultural soil and the native soil. Welch's t-tests followed by Bonferroni (*P*<0.05) corrections were performed at Phylum (a) and Class level (b). Only Phyla and Classes differentially abundant are shown.

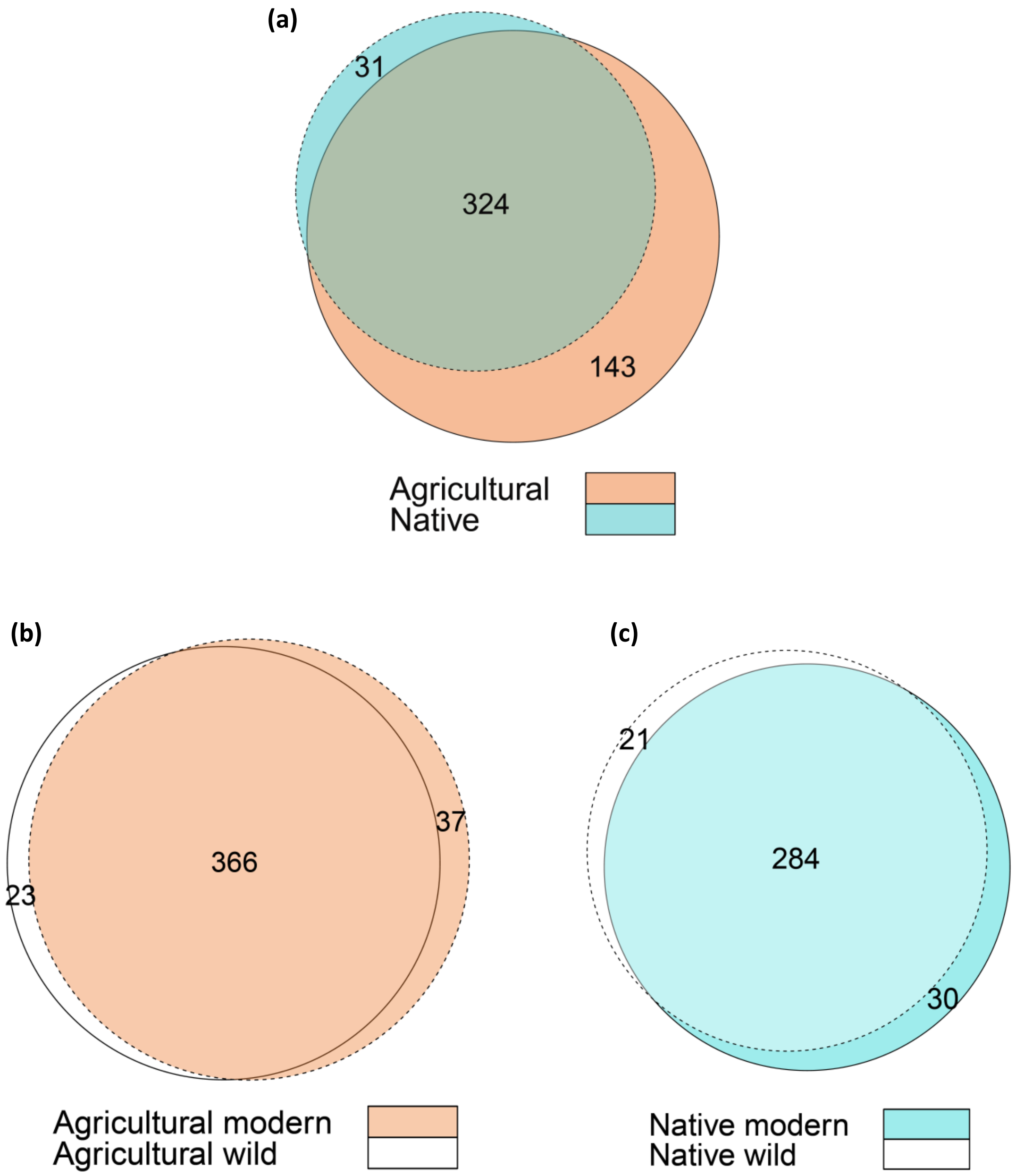

Figure S7. The enriched and depleted bacteria in the rhizosphere of wild and modern common bean grown in native and agricultural soils. Area-proportional Euler diagrams were built to depict the exclusive and the shared genera. (a) Number of genera shared between agricultural and native soils is depicted within the intersection while the number of genera exclusive to each soil type can be seen out of the intersection zone. The genera exclusive to the native soil are visible in the cyan colored area, while genera exclusive to the agricultural soil are visible in the dark orange colored area. (b) Euler diagram between modern accessions and wild accessions in the agricultural soil. The genera exclusive to the modern accessions is visible in the dark orange colored area, while genera exclusive to the wild accessions is visible in the white area. (c) Euler diagram between modern accessions and wild accessions in the native soil. The genera exclusive to the modern accessions are visible in the cyan colored area, while genera exclusive to the wild accessions are visible in the white area.

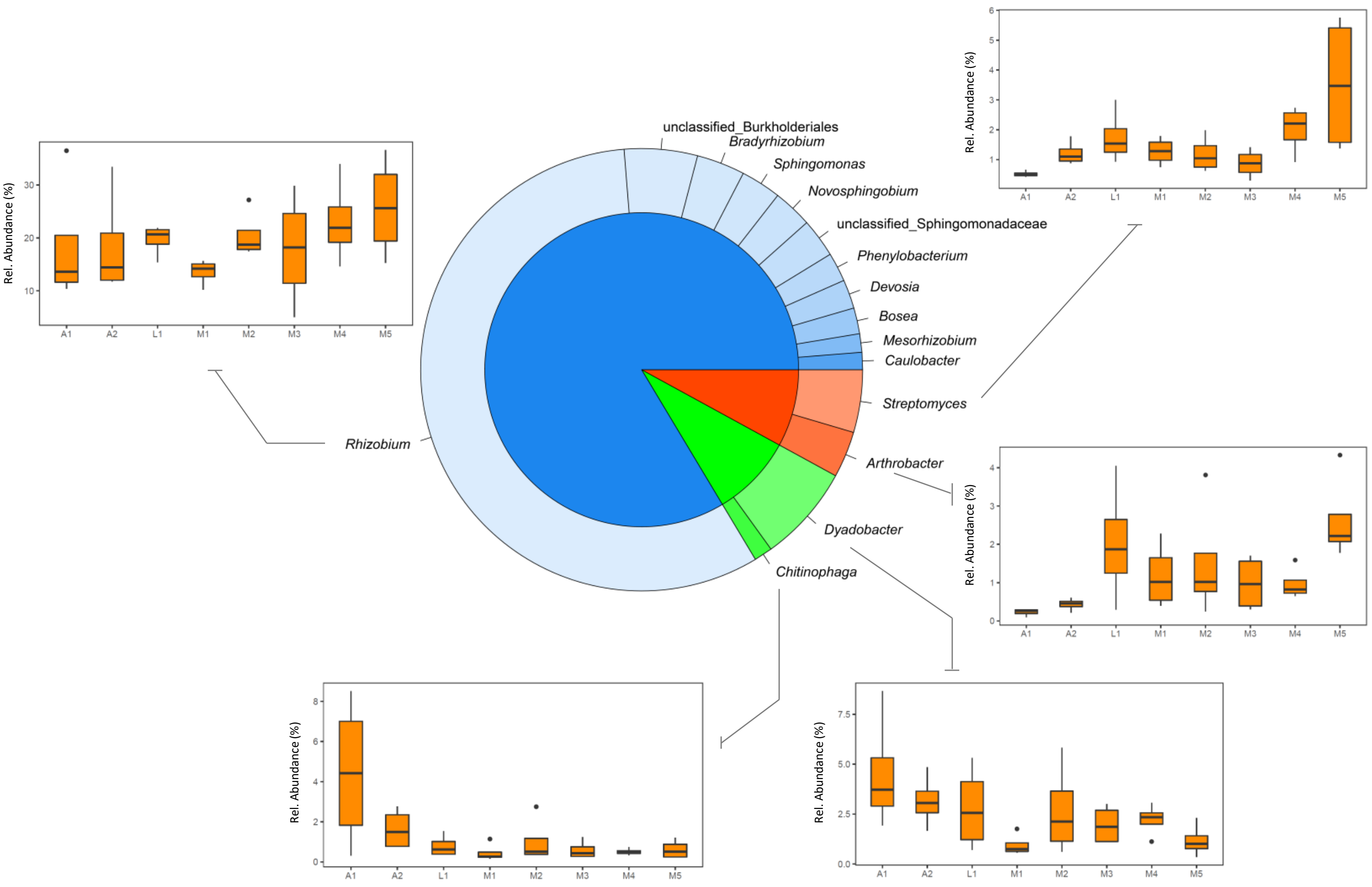

Figure S8. Core microbiome in the rhizosphere of common bean grown in agricultural soil. The different portions within the inner pie chart represent the bacterial phyla. The outer donut plot represents the genera that are part of the core. The size of the different pie and donut portions represents the contribution of each phylum/genus to the total relative abundance. Satellite box plots depict the relative abundance of selected genera by accession in the agricultural soil.

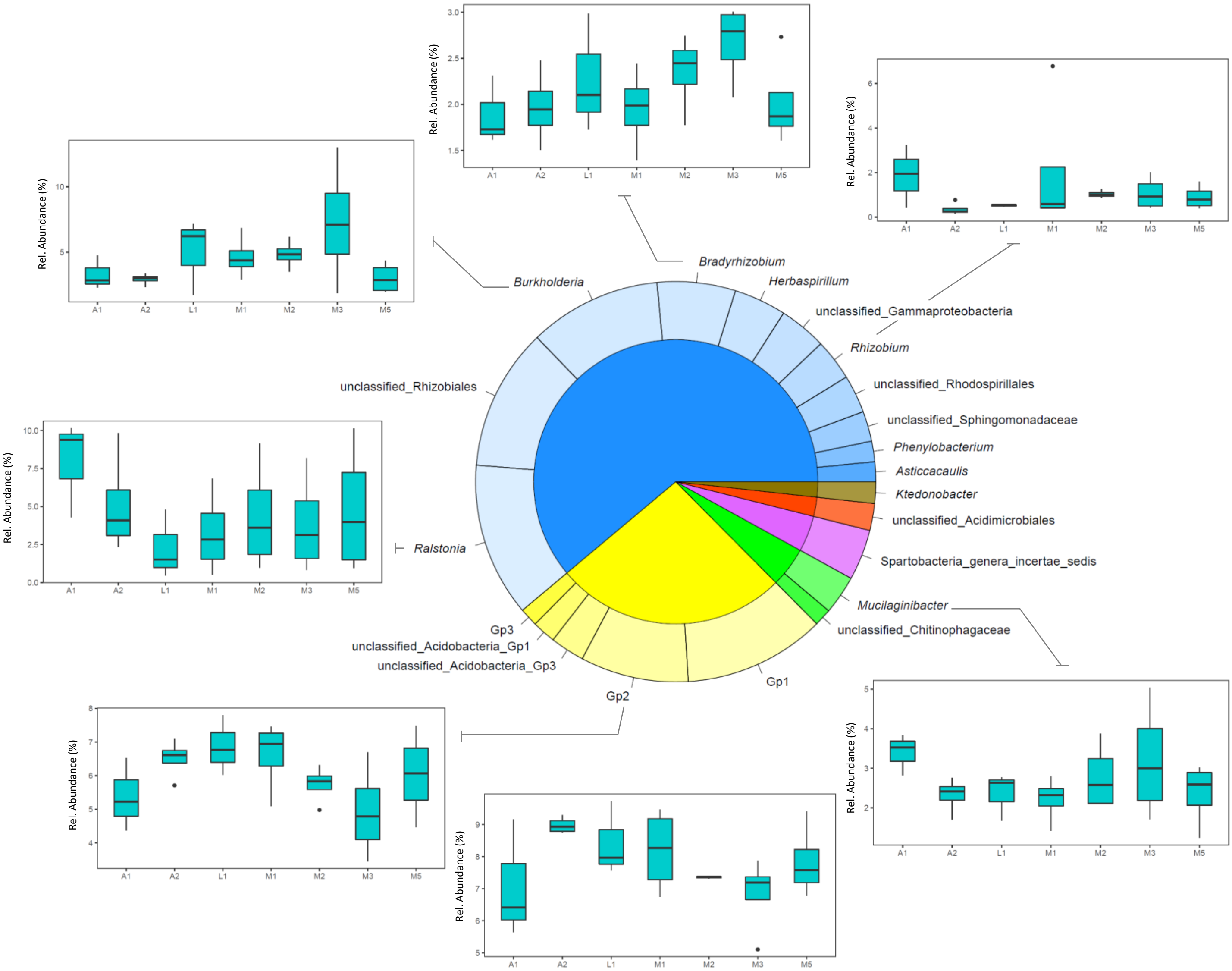

Figure S9. Core microbiome in the rhizosphere of common bean grown in native soil. The different portions within the inner pie chart represent the bacterial phyla. The outer donut plot represents the genera that are part of the core. The size of the different pie and donut portions represents the contribution of each phylum/genus to the total relative abundance. Satellite box plots depict the relative abundance of selected genera by accession in the native soil.

Supplementary tables

Table S1. Sequence characteristics obtained in the agricultural soil. In the table the raw read and after processing read counts are presented as well as quality characteristics of the raw reads.

Soil type	Sample	Total Bases	Raw read count	GC (%)	Q20 (%)	Q30 (%)	Processed read count
Agricultural	A1_1	63363676	211312	54.71	86.63	74.13	56996
Agricultural	A1_2	78985945	263296	55.18	87.15	75.19	77864
Agricultural	A1_3	69257541	231142	54.3	87.72	76.1	70601
Agricultural	A1_4	72760654	242636	54.82	87.79	76.02	69084
Agricultural	A2_1	69773872	232626	55.02	86.96	75.01	67646
Agricultural	A2_2	58210761	194134	54.35	88.36	76.93	60724
Agricultural	A2_3	76327328	254550	54.79	87.61	75.93	75603
Agricultural	A2_4	70190462	234008	55.13	86.55	74.44	67151
Agricultural	L1_1	72057959	240260	54.87	87.57	75.82	72136
Agricultural	L1_2	64508620	215002	55.81	87.01	74.95	63095
Agricultural	L1_3	68590650	228646	55.27	87.21	75.28	66964
Agricultural	L1_4	71258796	237562	55.41	87.34	75.6	72020
Agricultural	M1_1	71348421	237868	55.22	88	76.53	71859
Agricultural	M1_2	65375690	217894	55.94	86.24	73.77	58752
Agricultural	M1_3	67841335	226132	55.83	87.02	74.98	63630
Agricultural	M1_4	65532781	218424	55.57	86.86	74.81	63673
Agricultural	M2_1	67221432	224064	55.6	87.34	75.58	67676
Agricultural	M2_2	62678239	209020	55.15	87.26	75.32	61303
Agricultural	M2_3	66567882	221854	55.88	87.19	75.32	67063
Agricultural	M2_4	60348755	201286	54.78	87.97	76.36	61242
Agricultural	M3_1	59335191	197832	55.59	87.46	75.89	60023
Agricultural	M3_2	68861207	229586	55.1	88.35	76.94	70813
Agricultural	M3_3	68907707	229716	55.49	87.93	76.19	71553
Agricultural	M3_4	68367576	227898	55.67	87.18	75.22	68092
Agricultural	M4_1	74886328	249604	55.37	87.99	76.57	79568
Agricultural	M4_2	74054373	246918	55.4	87.51	75.82	73404
Agricultural	M4_3	74769091	249182	55.69	87.19	75.23	79061
Agricultural	M4_4	66904055	223064	55.4	87.95	76.29	69317
Agricultural	M5_1	62075633	206888	55.89	86.95	74.93	60638
Agricultural	M5_2	72482267	241660	55.61	87.82	76.35	74295
Agricultural	M5_3	61109216	203688	55.7	87.48	75.84	62822
Agricultural	M5_4	71365327	237914	55.51	87.73	76.17	72160
Agricultural	Bulk_soil_1	61607085	205346	56.88	86.33	73.98	59869
Agricultural	Bulk_soil_2	65310513	217716	56.66	85.85	73.09	58020
Agricultural	Bulk_soil_3	55162959	183890	56.6	86.69	74.51	51738
Agricultural	Bulk_soil_4	60880405	202966	56.4	86.27	73.96	55876

Table S1 (Continuation). Sequence characteristics of samples obtained in the native soil. In the table the raw read and after processing read counts are presented as well as quality characteristics of the raw reads.

Soil type	Sample	Total Bases	Raw read count	GC (%)	Q20 (%)	Q30 (%)	Processed read count
Native	A1_1	69790674	232642	55.48	87.61	75.83	68643
Native	A1_2	59019732	196740	55.3	87.94	76.32	59343
Native	A1_3	64931567	216392	56.16	86.45	73.82	60661
Native	A2_1	57934044	193128	56.32	86.58	74.47	55570
Native	A2_2	61055744	203522	56.51	87.04	74.95	60234
Native	A2_3	67056980	223502	55.93	86.85	74.57	63884
Native	A2_4	58518207	195056	55.75	87.08	75.01	56197
Native	L1_1	65521365	218470	56	87.59	75.83	65232
Native	L1_2	64785862	215952	55.81	87.13	75.17	65472
Native	L1_3	71386826	238006	56.23	87.42	75.58	70322
Native	M1_1	63411035	211380	55.54	87.88	76.22	62462
Native	M1_2	62074225	206932	56.22	87.78	76.08	63354
Native	M1_3	71515302	238420	56.3	86.88	74.78	68664
Native	M1_4	54123563	180468	55.92	87.98	76.29	55091
Native	M2_1	67140790	223824	56.07	87.71	76.01	67260
Native	M2_2	66608745	222070	56.26	87.66	75.92	65336
Native	M2_3	58726189	195734	56.03	86.89	74.83	56402
Native	M2_4	70057132	233546	55.9	87.05	75.05	69406
Native	M3_1	63700948	212352	55.9	87.25	75.32	63248
Native	M3_2	58356921	194520	56.26	86.7	74.5	55418
Native	M3_3	64816913	216052	56.18	86.53	74.28	60174
Native	M3_4	52757955	175792	55.73	85.03	71.51	45340
Native	M4_1	61945280	206504	55.92	87.14	75.06	60709
Native	M4_2	66279461	220998	55.67	87.04	74.99	63633
Native	M4_3	62281114	207616	55.92	87.18	75.18	59404
Native	M4_4	52537073	175068	56.84	82.13	67.37	35547
Native	Bulk_soil_1	51904334	173074	57.05	86.92	74.74	50134
Native	Bulk_soil_2	57678950	192304	57.01	86.5	74.18	54399
Native	Bulk_soil_3	64011567	213402	56.82	85.94	73.51	59298
Native	Bulk_soil_4	61453027	204890	56.77	86.45	74.16	57379

Table S2. AIC values for six rank abundance distribution models to test niche neutral models in native soil samples. The lowest AIC (Akaike Information Criterion) value for each sample represents the best fit model which is shown in bold.

	AIC					
**Sample**	**Broken-stick**	**Pre-Emption**	**Log-Normal**	**Zipf**	**Zipf-Mandelbrot**	**ZSM**
A1_1	109193.1	82138.6	**16519.6**	23002.6	22313	27723.2
A1_2	94184.6	69323.1	**12489.5**	17758.8	15560.7	25791.4
A1_3	75917.1	53531.6	**12942.3**	22601.2	nd	31087.8
A2_1	63993.3	46570.4	**13574.7**	22370.6	nd	29473.8
A2_2	71558.9	51409.5	**13565.3**	22980.2	nd	29706
A2_3	97215	73862	**16099**	22244	21654	27916.2
A2_4	67463.6	47477.7	**12704.8**	21889.1	nd	28406.8
L1_1	84656.2	57675.6	**13392.4**	24235.9	nd	29305.8
L1_2	76243.7	53640.4	**13452.2**	24314.4	nd	31883.8
L1_3	83374	53282	**14091**	28556	nd	28726.4
M1_1	102404	78828.6	**15112.9**	18962.9	17328.2	30968.2
M1_2	76043.8	51113.2	**12713**	23954.3	nd	30633.6
M1_3	86084.7	58044.4	**14829**	27170	nd	31285
M1_4	63313.4	45584.3	**12505.8**	21131.8	nd	28744.8
M2_1	81233.1	53271.9	**14479.4**	27657.3	nd	28957.2
M2_2	70881	47165.9	**14569**	27979.2	nd	31003.2
M2_3	82526.2	59967.7	**12385.5**	18537.5	nd	26264.8
M2_4	95319.6	64444	**12840.3**	23598.4	nd	27933.6
M3_1	83007.8	55441.1	**13188.9**	24134.3	nd	29499.2
M3_2	80158.3	60099.8	**13045.7**	18270.1	16701.8	29880.6
M3_3	82194.7	59553.1	**13634.7**	22082.8	nd	33823
M3_4	69381	50489	**10947.5**	15735.1	12638.3	25404.7
M4_1	65597.2	45892.6	**13894.5**	25206.3	nd	33552.8
M4_2	98371.9	75718.3	**16064.2**	21553.6	21041.2	29379
M4_3	75332.4	54770.3	**13519.5**	22214.1	17947.7	28705.4
M4_4	38329.1	29292.2	**11450.2**	17028.1	nd	27612.2
Bulk_soil_1	61291.3	41596.6	**12405.9**	21171.8	nd	24761.1
Bulk_soil_2	70455.2	45471.4	**12031.8**	21858.8	nd	24218.5
Bulk_soil_3	72354.2	47488.2	**12155.4**	22969	nd	25293
Bulk_soil_4	70091.6	45407.7	**12925**	24283.3	nd	25246.2

Table S3. Co-occurrence network properties of 16S rDNA rhizobacterial reads of common bean rhizosphere in agricultural and native soil

Network properties	Common bean Agricultural soil	Common bean Native soil
Number of nodes[a]	63	89
Number of edges[b]	61	176
Positive edges[c]	60	158
Negative edges[d]	1	18
Modularity	0.822	0.584
Number of communities	17	13
Network diameter	11	11
Average path length	3.87	4.06
Average degree	0.968	1.978
Average clustering coefficient	0.5	0.551

[a]Bacterial taxa (genus level) with at least one significant (P<0.01) and strong (SparCC > 0.8 or < -0.8) correlation.
[b]Number of connections/correlations obtained by SparCC analysis;
[c]SparCC positive correlation (> 0.8 with P < 0.01);
[d]SparCC negative correlation (< -0.8 with P < 0.01)
